# Reductive Hydrogenation of Sulfido-Bridged Tantalum Alkyl Complexes: A Mechanistic Insight

**DOI:** 10.1021/acs.inorgchem.3c00043

**Published:** 2023-06-15

**Authors:** Jorge J. Carbó, Manuel Gómez, Cristina Hernández-Prieto, Alberto Hernán-Gómez, Avelino Martín, Miguel Mena, Jordi Puiggalí-Jou, Josep M. Ricart, Cristina Santamaría

**Affiliations:** † Departamento de Química Orgánica y Química Inorgánica, Instituto de Investigación Química “Andrés M. del Río” (IQAR), Universidad de Alcalá, Campus Universitario, E-28805 Alcalá de Henares, Madrid, Spain; ‡ Departament de Química Física i Inorgànica, Universitat Rovira i Virgili, C/ Marcel.lí Domingo, s/n, 43007 Tarragona, Spain

## Abstract

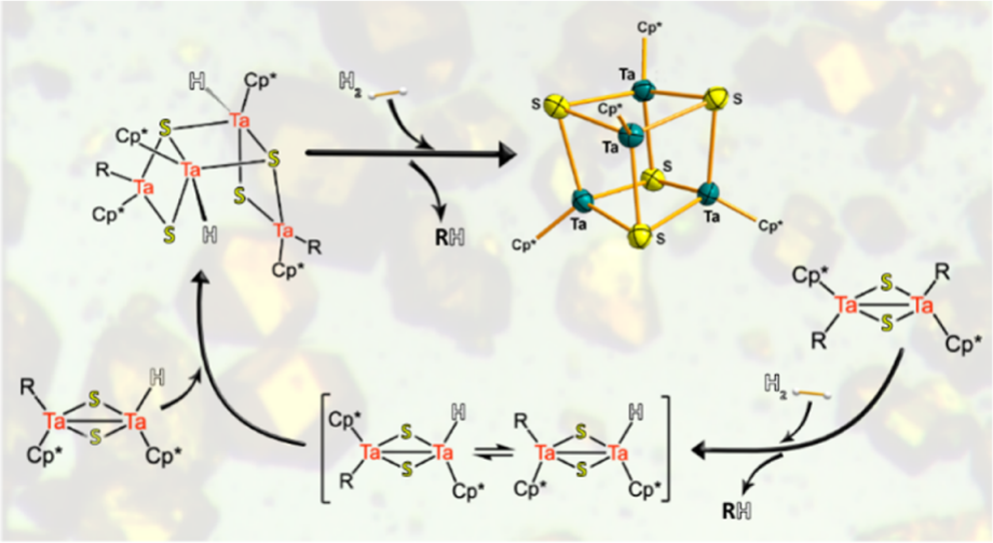

Hydrogenolysis of a series of alkyl sulfido-bridged tantalum(IV) dinuclear complexes [Ta(η^5^-C_5_Me_5_)R(μ-S)]_2_ [R = Me, *n*Bu (**1**), Et, CH_2_SiMe_3_, C_3_H_5_, Ph, CH_2_Ph (**2**), *p*-MeC_6_H_4_CH_2_ (**3**)] has led quantitatively to the Ta(III) tetrametallic sulfide cluster [Ta(η^5^-C_5_Me_5_)(μ_3_-S)]_4_ (**4**) along with the corresponding alkane. Mechanistic information for the formation of the unique low-valent tetrametallic compound **4** was gathered by hydrogenation of the phenyl-substituted precursor [Ta(η^5^-C_5_Me_5_)Ph(μ-S)]_2_, which proceeds through a stepwise hydrogenation process, disclosing the formation of the intermediate tetranuclear hydride sulfide [Ta_2_(η^5^-C_5_Me_5_)_2_(H)Ph(μ-S)(μ_3_-S)]_2_ (**5**). Extending our studies toward tantalum alkyl precursors containing functional groups susceptible to hydrogenation, such as the allyl-and benzyl-substituted compounds [Ta(η^5^-C_5_Me_5_)(η^3^-C_3_H_5_)(μ-S)]_2_ and [Ta(η^5^-C_5_Me_5_)(CH_2_Ph)(μ-S)]_2_ (**2**), enables alternative reaction pathways en route to the formation of **4**. In the former case, the dimetallic system undergoes selective hydrogenation of the unsaturated allyl moiety, forming the asymmetric complex [{Ta(η^5^-C_5_Me_5_)(η^3^-C_3_H_5_)}(μ-S)_2_{Ta(η^5^-C_5_Me_5_)(C_3_H_7_)}] (**6**) with only one propyl fragment. Species **2**, in addition to the hydrogenation of one benzyl fragment and concomitant toluene release, also undergoes partial hydrogenation and dearomatization of the phenyl ring on the vicinal benzyl unity to give a η^5^-cyclohexadienyl complex [Ta_2_(η^5^-C_5_Me_5_)_2_(μ-CH_2_C_6_H_6_)(μ-S)_2_] (**7**). The mechanistic implications of the latter hydrogenation process are discussed by means of DFT calculations.

## Abstract 2

Hydrogenation of a series of dimetallic alkyl/aryl/allyl sulfide-bridge complexes of tantalum, [Ta(η^5^-C_5_Me_5_)R(μ-S)]_2_, led to the isolation of the low-valent Ta(III) tetrametallic sulfur cube-type species [Ta(η^5^-C_5_Me_5_)(μ_3_-S)]_4_. Partial hydrogenolysis using phenyl, allyl, and benzyl moieties reveals plausible reaction pathways to the cube-type species. DFT studies confirm the low oxidation states of the synthesized compounds.

## Introduction

Hydrogenolysis of early transition metal alkyl compounds is a convenient methodology for the formation of low-valent transition metals via reductive elimination.^1−4^ Thus, the hydride route generates as the only byproduct highly volatile alkanes, which are easily removable,^5^ and avoids the use of external reductants,^6^ which very often produce undesirable side products as inorganic salts or over-reduced species. Remarkable examples of these reactions report the transformation of simple metal alkyl precursors into highly valuable low-valent species capable of mediating challenging transformations. In the field of small-molecule activation, Hou et al. reported how the hydrogenolysis of a C_5_Me_4_SiMe_3_-ligated titanium trialkyl compound leads to the formation of a trinuclear heptahydride complex with ability to promote the C–C bond cleavage of benzene,^7^ as well as the splitting and hydrogenation of N_2_.^8^ The same Hou also evidenced that these reactions can be extended to group 6 as treatment of a chromium alkyl species supported by a C_5_Me_4_SiMe_3_ ligand with H_2_ in the presence of N_2_ provides a tetranuclear diimide/dihydride complex [(Cp′Cr)_4_(μ_3_-NH)_2_(μ_3_-H)_2_] (Cp′ = C_5_Me_4_SiMe_3_), in which dinitrogen was reduced to NH^2–^ fragments.^9^ Within group 5, Fryzuk^10,11^ explored the hydrogenation of [(^Si^NPN)TaMe_3_] (^Si^NPN = PhP(CH_2_SiMe_2_NPh)_2_^2–^), which resulted in the formation of the dinuclear tantalum hydride species [{(NPN)Ta}_2_(μ-H)_4_]. Notably, the latter compound, besides mediating the fixation and functionalizing dinitrogen,^2,10−12^ also activates other small molecules such as CO_2_,^13^ CO,^14^ CS_2_,^15^ and N_2_H_4_.^16^ The versatility of this dimetallic system motivated the preparation and hydrogenation of other tantalum alkyl complexes with modified NPN as ancillary ligands.^17,18^ Even though these investigations provided new dimeric Ta species bridged by three hydride fragments, the generated products were unreactive toward small molecules, highlighting the major role played by the ancillary ligand in dictating the reactivity of the generated tantalum hydrides. Similarly, it is expected that modifying the fragments susceptible to hydrogenolysis will result in new and exciting outcomes. However, a systematic study of the hydrogenolysis of tantalum compounds bearing different reactive fragments with hydrogen has not been reported.

Recent efforts in our laboratory have focused on the synthesis of dinuclear sulfido-bridged tantalum complexes containing cyclopentadienyl as an ancillary ligand. This work resulted in the isolation of a series of bimetallic complexes of type [Ta(η^5^-C_5_Me_5_)R(μ-S)]_2_ (R = Cl, Me, Et, CH_2_SiMe_3,_ C_3_H_5_, and Ph),^19^ which possess potentially reactive M–C bonds. Building on these results, herein, we report the hydrogenolysis of the previous series of dimeric tantalum alkyl precursors, which results in the formation of a tetranuclear Ta(III) species. Replacement of the alkyl substituents by aryl, benzyl, and allyl fragments allows the isolation of different reaction intermediates, which is indicative of the existence of multiple reaction pathways in the hydrogenation reactions toward the formation of the final tetranuclear Ta(III) product. In addition, DFT calculations provide insight into the electronic structure of the tetranuclear Ta(III) species and the reaction mechanism of hydrogenolysis of Ta–alkyl bonds in dimetallic complexes.

## Results and Discussion

### Hydrogenation of Sulfido-Dialkyl Tantalum Complexes

**Scheme 1:**
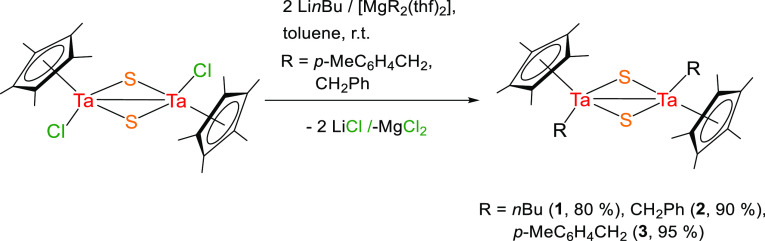
Synthesis of the Bimetallic Sulfide Complexes **1–3**

Aiming to investigate the influence of the alkyl fragment on the hydrogenation processes, we first extended the family of dinuclear tantalum sulfido complexes previously synthesized.^19^ Compounds [Ta(η^5^-C_5_Me_5_)R(μ-S)]_2_ [R = *n*Bu (**1**) CH_2_Ph (**2**), *p*-MeC_6_H_4_CH_2_ (**3**)] were prepared by the reaction of the dimetallic chloride species [Ta(η^5^-C_5_Me_5_)Cl(μ-S)]_2_ with the corresponding alkylating reagent, [MgR_2_(thf)_2_] (R = CH_2_Ph, *p*-MeC_6_H_4_CH_2_) or Li*n*Bu, at room temperature in toluene or hexane (Scheme 1). The characterization of compounds **1–3** by multinuclear NMR spectroscopy (see Experimental Section) confirms a dinuclear arrangement in which the cyclopentadienyl groups adopt a trans configuration, similar to the parent chloroderivative and the previously reported tantalum alkyl compounds.^19^

**Scheme 2:**
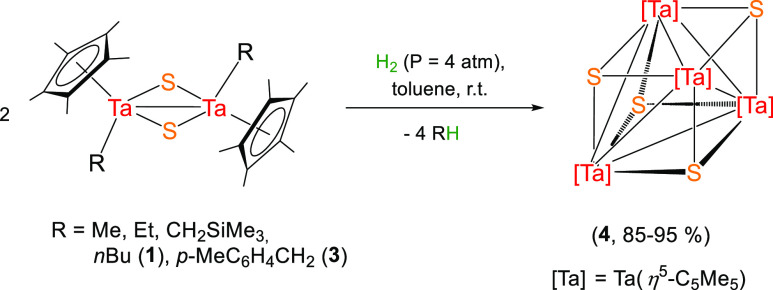
Synthesis of the Tantalum Sulfide Cube-Type Complex **4**

Hydrogenolysis of the species [Ta(η^5^-C_5_Me_5_)R(μ-S)]_2_ [R = Me, Et, CH_2_SiMe_3_, *n*Bu (**1**), and *p*-MeC_6_H_4_CH_2_ (**3**)] under analogous conditions (4 atm of H_2_, 24–48 h., room temperature) was first investigated. In all cases, upon H_2_ splitting, the dimeric species lead to the formation of the tetranuclear tantalum(III) compound [Ta(η^5^-C_5_Me_5_)(μ_3_-S)]_4_ (**4**), arranged in a cubane-type structure, as outlined in Scheme 2. Despite the simple structural nature of this compound, its formation should involve multiple steps: hydrogenolysis, converting the Ta–C bonds into Ta–H, combined with reductive elimination, and a dimerization step. However, monitoring these reactions by ^1^H NMR only displays the formation of the corresponding alkane compound and one singlet at 2.20 ppm assigned to the η^5^-C_5_Me_5_ of the symmetrical species **4**.

**Figure 1:**
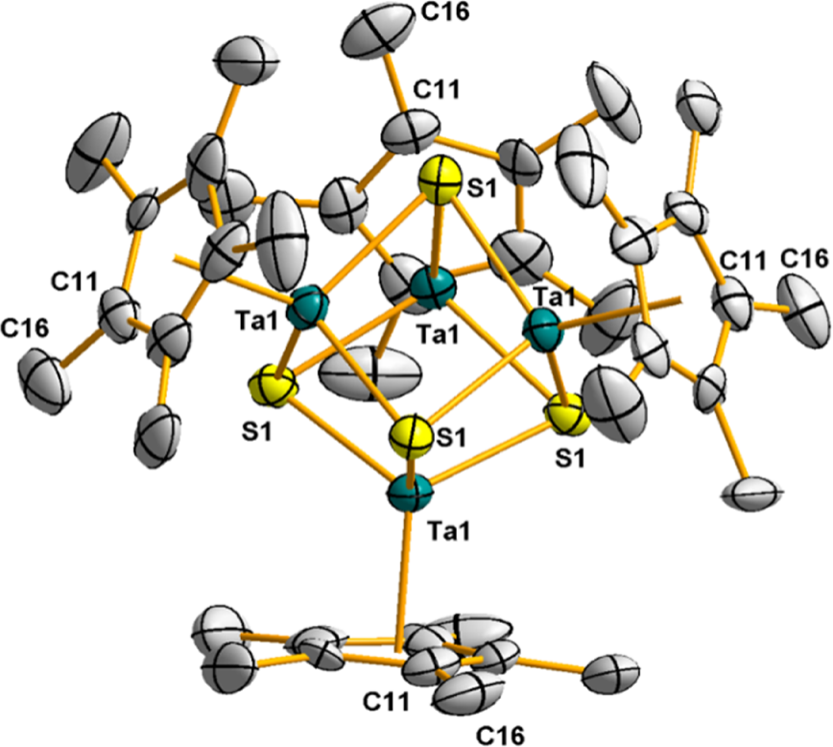
Molecular structure of **4**. Thermal ellipsoids are at 50% probability. Hydrogen atoms and Ta–Ta metal bonds have been omitted for clarity. Selected averaged lengths (Å) and angles (°): Ta–S 2.413(5), Ta–Ta, 2.98(1), Ta–S–Ta 76.2(5), S–Ta–S 102.3(8), and Ta–Ta–Ta 60.0(4).

The molecular structure of **4**, see Figure 1, reveals a homometallic M_4_S_4_ distorted cube that can be described as two interpenetrated tetrahedra of Ta_4_ and S_4_, where the sulfur atoms cap the four faces of the Ta_4_ tetrahedron. Additionally, each tantalum atom is linked to a pentamethylcyclopentadienyl ligand in a three-legged piano stool geometry. The bond distances Ta–S of 2.413(5) Å are slightly longer than those found for the dinuclear precursors [Ta(η^5^-C_5_Me_5_)R(μ-S)]_2_ (R = Cl, Me, Ph, and av 2.32(3) Å),^19^ but similar to those observed for the trinuclear complexes [Ta_3_(η^5^-C_5_Me_5_)_3–*n*_Cl_3+*n*_(μ_3_-Cl)(μ-S)_3_(μ_3_-S)] [*n* = 0, 1; 2.413(3)–2.546(3)].^19^ Likewise, the intermetallic distance (Ta···Ta = 2.98(1) Å)^20^ in **4** is marginally longer than those registered for the dinuclear compounds (2.918(1)–2.951(1) Å), but shorter than those found for the trinuclear species (3.402(1)–3.545(1) Å), or the cube-type derivatives [TaCl(NR)py(μ_3_-S)]_4_.^21^

**Figure 2:**
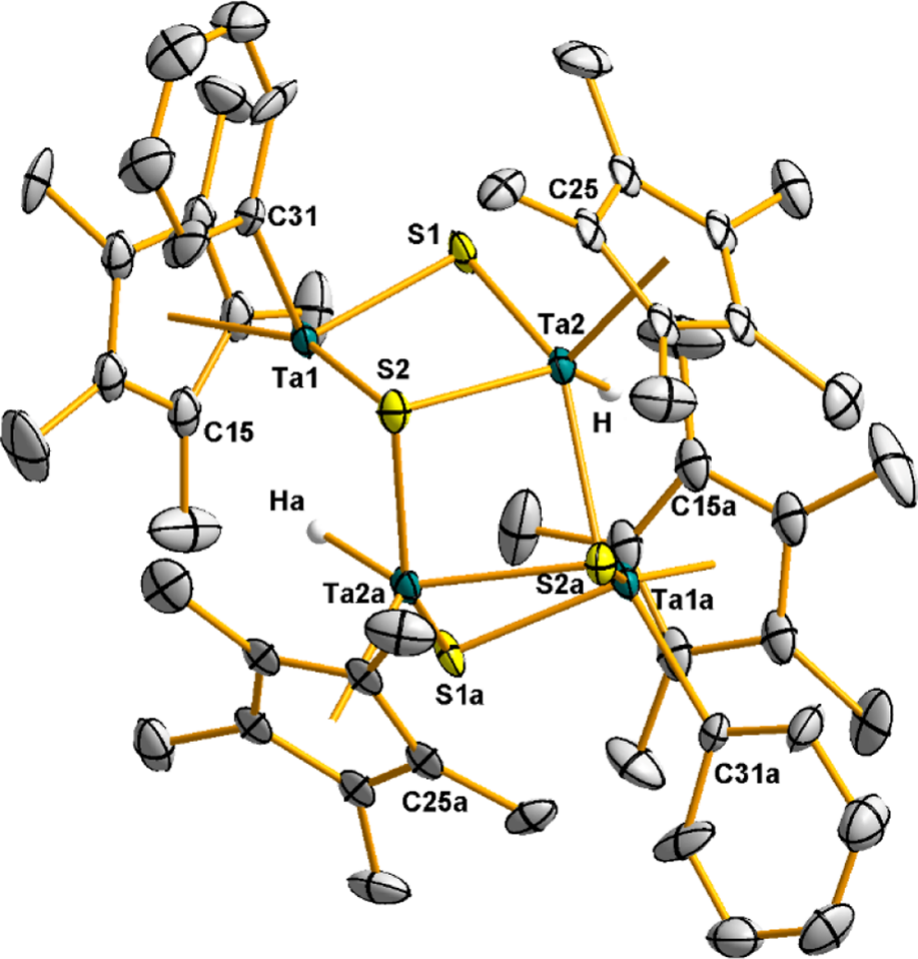
Molecular structure of **5**. Thermal ellipsoids are at 50% probability. Hydrogen atoms of η^5^-C_5_Me_5_ and phenyl groups are omitted for clarity. Selected averaged lengths (Å) and angles (°): Ta···Ta 3.20(4), Ta2–H 1.65, Ta1–C31 2.24(1), Ta1–S1 2.285(3), Ta1–S2 2.513(4), Ta2–S1 2.440(4), Ta2–S2 2.408(3), Ta2–S2a 2.450(4); S1–Ta2–H 68.0, S2–Ta2–H 137.0, S2a–Ta2–H 90.3, S1–Ta1–S2 96.2(1), S1–Ta2–S2a 141.4(1), S2–Ta2–S1 95.0(1), S2–Ta2–S2a 79.3(1), and Ta–S–Ta 82(2).

**Scheme 3:**
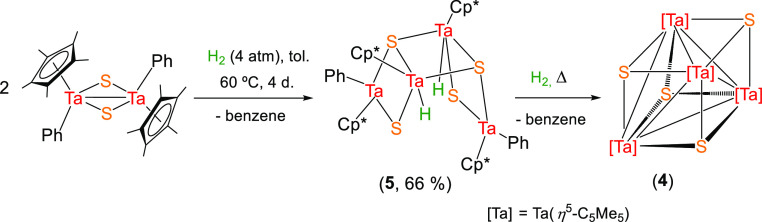
Synthesis of the Tetranuclear Sulfide Complex **5**

Intrigued by the intricate process of the formation of compound **4**, we explored the hydrogenolysis of [Ta(η^5^-C_5_Me_5_)Ph(μ-S)]_2_ bearing a less basic phenyl moiety compared to previous alkyl fragments. Indeed, this process requires forcing the reaction conditions to 4 atm of H_2,_ 60 °C and 4 days to afford quantitatively the tetrametallic Ta(IV) hydride species [Ta_2_(η^5^-C_5_Me_5_)_2_(H)Ph(μ-S)(μ_3_-S)]_2_ (**5**), as determined by X-ray diffraction analysis (Scheme 3, Figure 2).^22^

Structurally, compound **5** displays a distorted ladder-type tricyclic arrangement formed by two sets of [Ta(η^5^-C_5_Me_5_)Ph] and [Ta(η^5^-C_5_Me_5_)H] fragments linked by four bridging sulfur atoms. This molecular arrangement suggests that the formation of **5** combines the hydrogenolysis of only one phenyl group of the dinuclear dialkyl precursors with a dimerization process. Similar molecular structures have been reported for the tetranuclear imido and sulfur-bridged tantalum compounds [Ta_4_(η^5^-C_5_H_5_)_2_(μ-Cl)(N^*t*^Bu)_4_py_2_(μ_3_-S)_2_(μ-S)_2_](C_5_H_5_).^21^ Further analysis of the molecular structure of **5** reveals that within each dimer, one of the tantalum atoms exhibits a three-legged piano-stool geometry with the cyclopentadienyl rings located on the apical positions and two sulfur atoms and one phenyl ligand occupying the basal apexes, while the vicinal tantalum exhibits a four-legged piano-stool with the cyclopentadienyl rings located on the apical positions and three sulfur atoms and one hydride ligand occupying the basal apexes. Although the position of the hydride atom in the diamagnetic compound 5 was determined in the difference Fourier map and its position refined, there is a slight uncertainty in this assignment due to the proximity of heavy tantalum and sulfur atoms that can overwhelm the small electron density of the H atom.

The presence of the hydride ligand is further confirmed by the observation of a band at 1633 cm^–1^ in the IR spectrum and a highly upfield resonance in the ^1^H NMR spectrum at −4.98 ppm. The latter delta value compares well with the data registered for other tantalum terminal hydride species such as [Ta(η^5^-C_5_Me_5_){η^5^-C_5_H_4_(SiMe_3_)_2_}H(CNR)] (R = 2,6-Me_2_C_6_H_3_NC, δ = −4.45).^23^ Supporting the idea that species **5** is an intermediate in the formation of compound **4**, when a toluene solution of **5** was exposed to a dihydrogen atmosphere over a longer period of time, it led to the formation of the cubane compound **4** (Scheme 3).

**Scheme 4:**
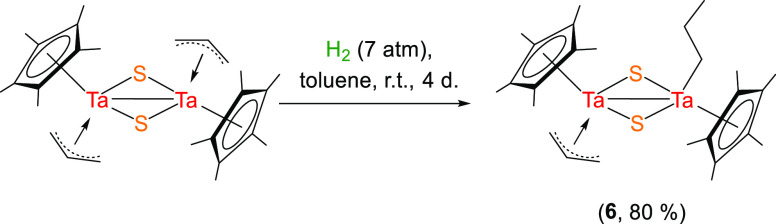
Synthesis of the Tantalum Allyl Propyl Complex **6**

Encouraged by the latter result, we next evaluated the influence of the alkyl substituent attached to tantalum on the hydrogenation outcome. Thus, the reaction of the allyl derivative [Ta(η^5^-C_5_Me_5_)(η^3^-C_3_H_5_)(μ-S)]_2_ with 7 atm of hydrogen at room temperature for 4 days resulted in the hydrogenation of only one of the two allyl fragments, leading to compound [{Ta(η^5^-C_5_Me_5_)(η^3^-C_3_H_5_)}(μ-S)_2_{Ta(η^5^-C_5_Me_5_)(C_3_H_7_)}] (**6**) with a propyl moiety (Scheme 4). Longer reaction times only resulted in the formation of the final product **4**, with no detection of any intermediate species.

A similar process can be found for the hydrogenolysis of the alkyne benzyl tantalum compound [(NPN*)Ta(BTA)(CH_2_Ph)] (NPN* = PhP(2-(*N*-mesityl)-5-Me-C_6_H_3_)_2_; BTA = bis(trimethylsilyl)acetylene) reported by Fryzuk, in which under controlled H_2_ pressure and short reaction times an alkene hydride tantalum species generated by partial hydrogenation is isolated.^18^

**Figure 3:**
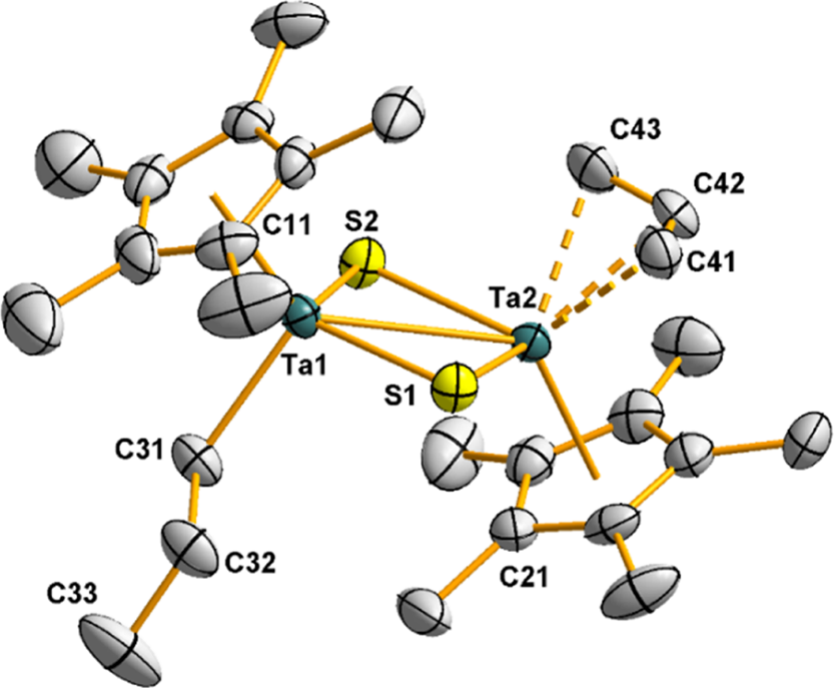
Molecular structure of **6**. Thermal ellipsoids are at 50% probability. Hydrogen atoms are omitted for clarity. Selected averaged lengths (Å) and angles (°): Ta1–S 2.288(1), C–C (allyl) 1.407(2), C–C (propyl) 1.53(2), Ta2–S 2.434(2), Ta1–C31 2.179(5), Ta2–C_allyl_ 2.33(1), Ta1–Ta2 3.033(1), Ta1–S1–Ta2 79.8(1), S1–Ta1–S2 104.4(1), S1–Ta2–S2 95.9(1), Ta1–S2–Ta2 79.9(1), C31–Ta1–S1 105.0(1), and C31–Ta1–S2 104.8(1).

The solid-state structure of complex **6**, determined by X-ray crystallography studies (Figure 3), reveals that the partial hydrogenation does not significantly modify the structural parameters of the core [Ta(η^5^-C_5_Me_5_)(μ-S)]_2_ compared to the similar bimetallic alkyl precursors.^19^ For instance, the Ta···Ta distance of 3.033(1) Å is close to those registered for the species [Ta(η^5^-C_5_Me_5_)R(μ-S)]_2_ [R = Me 2.929(1) Å, Ph 2.918(1) Å] and species with the Ta–Ta single bond in the oxidation state (IV).^24^

**Scheme 5:**
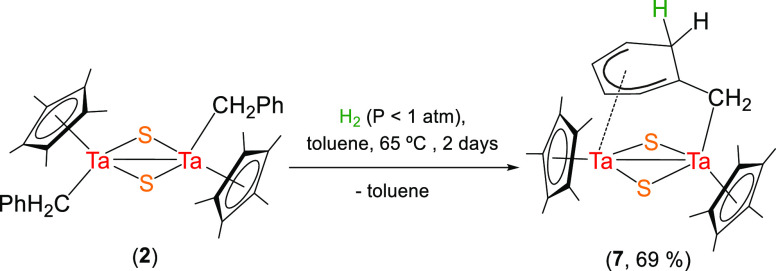
Hydrogenolysis of the Tantalum Dibenzyl Tantalum Complex **2**

A significantly different reactivity pattern was observed for the dibenzyl tantalum compound [Ta(η^5^-C_5_Me_5_)(CH_2_Ph)(μ-S)]_2_ (**2**), which reacts with H_2_ (<1 atm) at 65 °C in toluene solution to produce the hydrogenolysis of one alkyl moiety, while the second one is transformed into a cyclohexadienylmethylene fragment through a hydrogenation process, forming the species [Ta_2_(η^5^-C_5_Me_5_)_2_(μ-CH_2_C_6_H_6_)(μ-S)_2_] (**7**) (Scheme 5).

NMR spectroscopy for the diamagnetic complex **7** displays two signals at very different chemical shifts (δ = 2.05 and 1.76) due to the presence of two inequivalent pentamethylcyclopentadienyl ligands. The four methine protons of the cyclohexadienyl fragment resonate as multiplets at δ 5.00, 3.71, 2.99, and 2.27, while the two diastereotopic methylene units (cyclohexadienyl and Ta–CH_2_C_6_H_6_) were found as AX spin systems at δ 3.49, 2.56 (^2^*J* = 11.0 Hz) and at δ 0.25, 1.12 (^2^*J* = 11.0 Hz), respectively. Additionally, the ^13^C NMR spectrum exhibited two signals for these methylene groups at δ 29.2 and 65.7. Finally, we observed that compound **7** can be transformed into the tetrametallic tantalum(III) compound **4** by reaction with H_2_ (1 atm) at 70 °C for several days.

**Figure 4:**
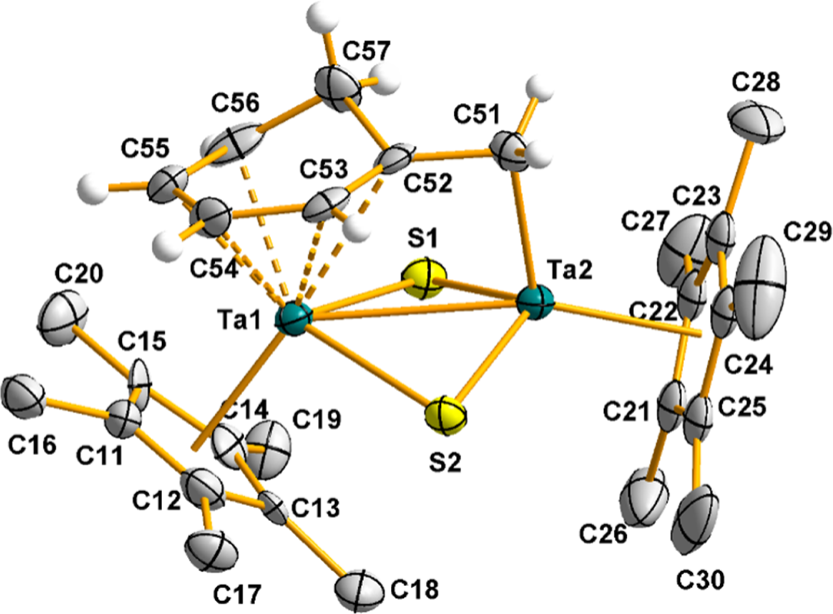
Molecular structure of **7**. Thermal ellipsoids are at 50% probability. The hydrogen atoms of the pentamethylcyclopentadienyl ligands are omitted for clarity. Selected averaged bond lengths (Å) and angles (°): Ta···Ta 2.988(2), Ta1–S1 2.524(3), Ta1–S2 2.590(3), Ta2–S1 2.275(3), Ta2–S2 2.274(3), Ta3–S3 2.578(3), Ta3–S4 2.551(3), Ta4–S3 2.269(3), Ta4–S4 2.276(3), Ta2–C51 2.18(1), Ta4–C61 2.19(1), S–Ta1/Ta3–S 90.3(3), S–Ta2/Ta4–S 106.0(1), and Ta–S–Ta 75.9(3).

The solid-state structure of one of the two crystallographic independent molecules of **7**, along with a selection of interatomic distances and angles, is depicted in Figure 4. This compound shows a dinuclear structure, in which two tantalum atoms are bridged by two sulfur atoms and a μ-CH_2_C_6_H_6_ fragment. The partial hydrogenation and dearomatization of the latter moiety are evidenced by the position of C57, which is located ≈0.55 Å above the plane formed by the C52–C56 atoms. Although both metal centers exhibit a three-legged piano-stool geometry, formed by a pentametylcyclopentadienyl ring and two sulfur atoms, the third position is differently occupied by a methylene group in the case of Ta2, and a η^5^-cyclohexadienyl moiety for Ta1. The bond distances from Ta1 to the carbon atoms of the η^5^-cyclohexadienyl fragment are in the range of 2.33(1)–2.51(1) Å, which is similar to the metrical parameters found for a related tantalum compound reported by Tilley,^25,26^ in which a η^5^-cyclohexadienyl fragment, also generated by hydrogenation of a phenyl ring, interacts with a vicinal Ta atom. The distance between the two metal centers (2.988(2) Å) is slightly longer than those observed in the alkyl precursors [Ta(η^5^-C_5_Me_5_)R(μ-S)]_2_ [R = Me 2.929(1) Å, Ph 2.918(1) Å], but still within the range for an intermetallic bonding interaction.^19^

### Computational Studies

**Figure 5:**
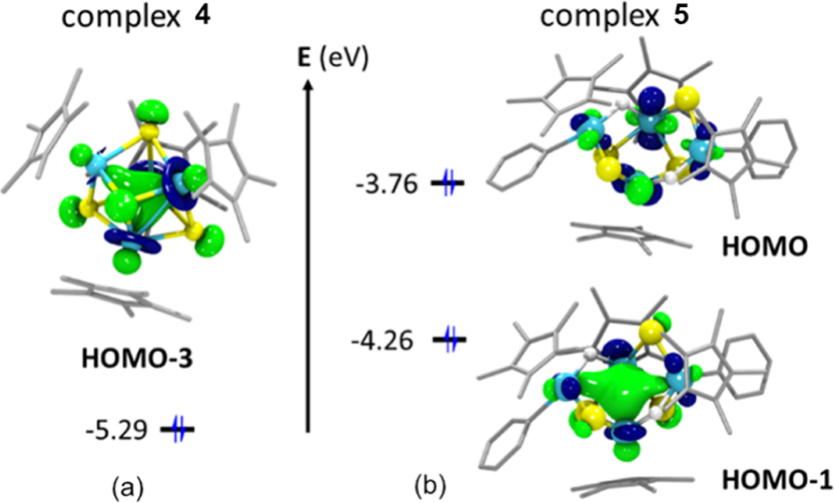
Frontier molecular orbitals of complexes **4** (a) and **5** (b).

Our initial analysis aimed to elucidate the bonding situation for the dinuclear parent compounds **1–3**, the tetranuclear [Ta(η^5^-C_5_Me_5_)(μ_3_-S)]_4_ (**4**), and [Ta_2_(η^5^-C_5_Me_5_)_2_(H)Ph(μ-S)(μ_3_-S)]_2_ (**5**). Similarly to our previous studies on the electronic configuration of dinuclear sulfide Ta(IV) species,^19,27^ compounds **1–3** display a HOMO orbital consisting of a σ-bonding combination between the d orbitals of the tantalum atoms, proving a σ bond between the two Ta(IV) centers (see Figure S28). For the cube-type structure **4**, we could identify four occupied molecular orbitals (from HOMO to HOMO – 3) based on tantalum d-type orbitals, which is a clear indication of the oxidation state III of tantalum atoms. The HOMO – 3 orbital (Figure 5a) is a bonding combination of atomic d-type orbitals at the four tantalum centers, indicating that in complex **4**, there is metal–metal interaction, although the four d-type electron pairs are delocalized over six possible Ta–Ta junctions. In line with canonical DFT orbital analysis, the computed Wiberg bond index (WBI) averaged for the six Ta–Ta interactions is 0.63, which is lower than that for dinuclear complex **2** (0.72). For complex **5,** the frontier molecular orbitals HOMO and HOMO – 1 are tantalum d-type orbitals of non-bonding and bonding nature, respectively (see Figure 5b). The bonding HOMO – 1 orbital is delocalized over the four tantalum centers, but with a higher contribution of the atomic orbitals at the two central tantalum atoms of the tricyclic arrangement ([Ta(η^5^-C_5_Me_5_)H] fragments). Delocalization of the electron pair reduces the bond order of the Ta–Ta bonding in the tetrametallic complex **5** (WBI = 0.45), with a crystallographic Ta–Ta distance of 3.20 Å, significantly longer than those found for the previously characterized dimetallic sulfide Ta(IV) complexes (ranging from 2.92 to 2.95 Å), for which a Ta–Ta σ bond was proposed.^19^

**Figure 6:**
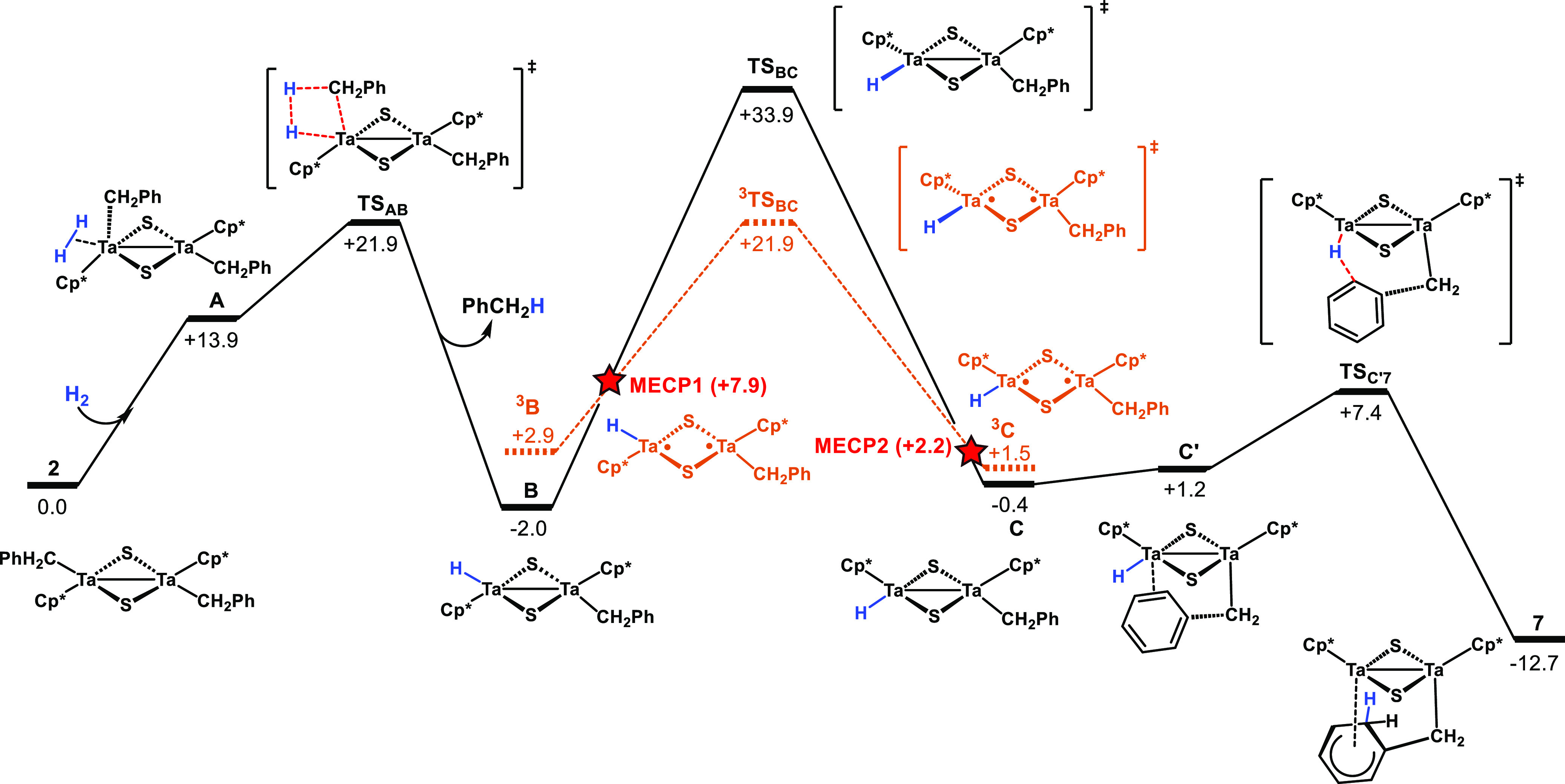
Gibbs free-energy profile (kcal·mol^–1^) for the hydrogenation process of the dibenzyl tantalum complex **2** to yield complex **7**. Dashed red lines denote the triplet state, and the red star stands for a minimum energy crossing point (MECP) between the singlet and the triplet potential energy surfaces.

Next, we focused our attention on the hydrogenolysis of Ta-alkyl compounds and computationally analyzed the mechanism of the reaction of [Ta_2_(η^5^-C_5_Me_5_)_2_(CH_2_Ph)_2_(μ-S)_2_] (**2**) with H_2_ to yield toluene and complex **7**, [Ta_2_(η^5^-C_5_Me_5_)_2_(μ-CH_2_C_6_H_6_)(μ-S)_2_]. The proposed mechanism (Figure 6) can be divided into three main stages: (i) toluene elimination by hydrogenation of one Ta-benzyl fragment resulting in a *trans* Ta(IV)-hydride Ta(IV)-benzyl intermediate, (ii) *trans*–*cis* isomerization bringing closer the hydride and benzyl ligands of both metal centers, and (iii) partial hydrogenation of the phenyl ring of the remaining benzyl ligand.

First, one of the Ta(IV) centers of complex **2** coordinates the H_2_ molecule to form the intermediate **A** (Figure 6). Then, H_2_ addition to the Ta–C bond of a benzyl fragment takes place, releasing toluene and leading to the Ta(IV) hydride complex [{Ta(η^5^-C_5_Me_5_)(H)}(μ-S)_2_{Ta(η^5^-C_5_Me_5_)(CH_2_Ph)}] (**B**), in which the hydride and the benzyl ligands are trans to each other. The computed, overall free-energy barrier for the H_2_ addition (**2** + H_2_ → **B** + toluene) is 21.9 kcal·mol^–1^, and the intermediate **B** is 2.0 kcal·mol^–1^ below the reactants. This indicates that the process is both kinetically and thermodynamically feasible.

Alternatively, by analogy with our previous study on the N=N bond cleavage by dinuclear hydride Ta(IV) complexes,^27^ we also evaluated the activation of H_2_ by oxidation of the Ta(IV)–Ta(IV) bond to yield the dihydride Ta(V) complex [Ta(η^5^-C_5_Me_5_)(H)(CH_2_Ph)(μ-S)]_2_. However, this path can be ruled out since its computed free energy (23.0 kcal·mol^–1^) is higher than that of the current transition state **TS**_**AB**_. A third alternative was explored for the addition of H_2_ across the Ta–S bond;^28−30^ however, it is also energetically disfavorable when compared with the free energy of the **TS**_**AB**_ structure (the resulting intermediate is 37.6 kcal·mol^–1^ above reactant **2**).

**Figure 7:**
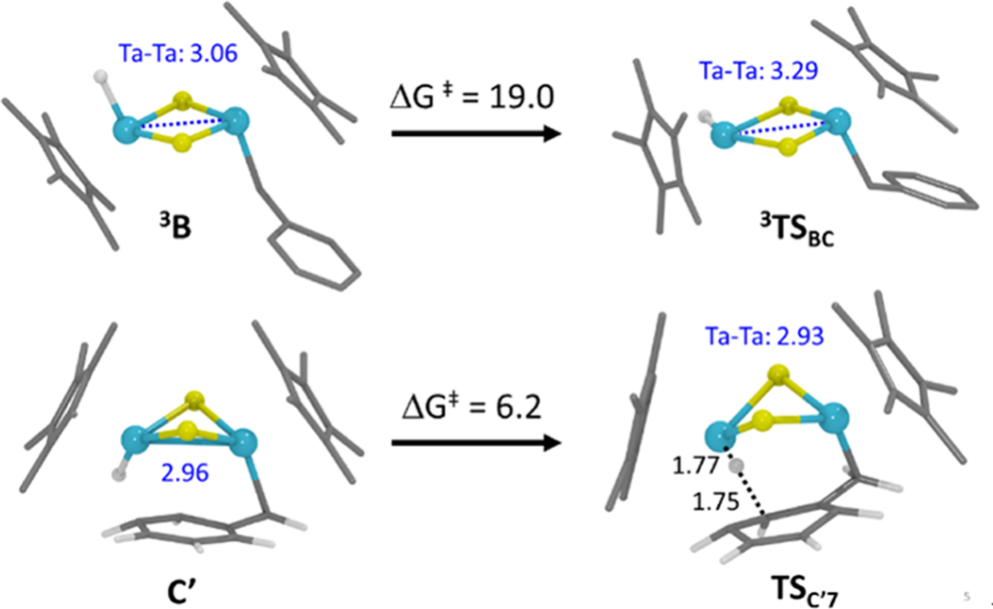
DFT structures of selected intermediates and transition states. Distances are given in Å and free energy barriers in kcal·mol^–1^.

In the next step of the mechanism, the intermediate **B** undergoes a *trans*-to-*cis* isomerization in order to place the hydride and the benzyl ligands on the same side of the [Ta_2_(μ-S)_2_] core (Figure 6), enabling the hydrogenation of the aromatic ring. Similar to our own previous studies for other dinuclear sulfide Ta(IV) alkyl compounds,^19^ calculations predict that the isomerization process is only slightly endergonic (+1.6 kcal·mol^–1^). For this process, two possible transition states with an electronic configuration of triplet or singlet are possible; the former reveals a lower free-energy barrier (19.0 kcal·mol^–1^ for triplet vs 35.9 kcal·mol^–1^ for singlet). The isomerization occurs by exchanging the positions of the hydride and the pentamethylcyclopentadienyl ligands via a square-planar [Ta(η^5^-C_5_Me_5_)(H)(μ-S)_2_] transition state (Figure 7). The excitation to the triplet state involves the cleavage of the Ta–Ta bond moving away the two tantalum fragments and reducing the cost of forming the square-planar geometry at the transition state. Thus, the reaction could hop from the singlet to the triplet potential energy surfaces, yielding the ^**3**^**B** intermediate. Then, this intermediate isomerizes to form the *cis* hydride–benzyl complex ^**3**^**C**, which hops back to the singlet energy surface, giving complex **C**. Overall, the spin crossing from the singlet to the triplet energy surface would yield a lower isomerization energy barrier (23.9 kcal·mol^–1^, from **B** to ^**3**^**TS**_**BC**_). We could find two MECP connecting **B** with ^**3**^**B** and ^**3**^**C** with **C** whose energy values are indicative of moderate activation barriers for the spin crossover processes, 9.9 and 0.7 kcal·mol^–1^, respectively. In addition, due to the influence of heavy tantalum atoms in the structure of MECP, the spin–orbit coupling is expected to be large, favoring the transition from the singlet to the triplet and back to the singlet surface. Alternative pathways were evaluated for this reaction step; nevertheless, they were discarded due to their prohibitive energetic barriers (see Figures S29 and S30).

Finally, the free rotation along the Ta–Csp^3^ bond of the benzyl ligand places the aromatic ring close to the hydride bound to the second tantalum center in the intermediate **C′**, leading to the hydrogenation of the aromatic ring at the ortho position. According to the experimental result, the computed free-energy barrier through the **TS**_**C′7**_ transition state is feasible (7.8 kcal·mol^–1^ from **C**), and the resulting final product **7** lies 12.7 kcal·mol^–1^ below the reactants. Interestingly, calculations suggest that this later hydrogenation step is reversible with a moderate reverse free-energy barrier of 20.1 kcal·mol^–1^ (from **7** to **TS**_**C7**_), lower than the overall free-energy barrier of the forward process, 23.9 kcal·mol^–1^ (from **B** to ^**3**^**TS**_**BC**_).

## Conclusions

We have shown that the hydrogenation of a series of dimetallic alkyl/aryl/allyl sulfide-bridge complexes of tantalum, [Ta(η^5^-C_5_Me_5_)R(μ-S)]_2_, led to the isolation of the low-valent Ta(III) tetrametallic sulfur cube-type species [Ta(η^5^-C_5_Me_5_)(μ_3_-S)]_4_. Furthermore, by judiciously selecting the fragment attached to tantalum, we have been able to isolate the intermediate species [Ta_2_(η^5^-C_5_Me_5_)_2_(H)Ph(μ-S)(μ_3_-S)]_2_ (**5**), in which partial hydrogenation and dimerization have occurred prior to the final formation of **4**. Alternatively, using allyl and benzyl moieties enables new routes toward the formation of species **4**, via partial hydrogenolysis. DFT analysis provides valuable information about the electronic configuration of the tetrametallic structures, confirming the oxidation state III for the cube-type structure [Ta(η^5^-C_5_Me_5_)(μ_3_-S)]_4_, and IV for compound [Ta_2_(η^5^-C_5_Me_5_)_2_(H)Ph(μ-S)(μ_3_-S)]_2_ (**5**). Additionally, our calculations suggest a route for the formation of the unexpected compound [Ta_2_(η^5^-C_5_Me_5_)_2_(μ-CH_2_C_6_H_6_)(μ-S)_2_] (**7**), in which the hydrogenolysis of the dimetallic compound [Ta(η^5^-C_5_Me_5_)(CH_2_Ph)(μ-S)]_2_ (**2**) proceeds through the heterolytic H_2_ addition to the Ta–benzyl bond, releasing toluene and generating a Ta-hydride intermediate. Then, the reaction evolves through an isomerization process that allows the Ta-hydrido group to hydrogenate the aromatic ring of the benzyl ligand. Finally, based on the detected reversibility between **7** and **TS**_**C7**_, in which the hydride shifts from the cyclohexadienyl ring to the metal, we envisage the use of these species as masked hydride tantalum compounds. Therefore, further efforts with this species will be directed toward analyzing the catalytic hydrogenation of unsaturated organic molecules.

## Experimental Section

### General Considerations

All manipulations were carried out under a dry argon atmosphere using Schlenk-tube and cannula techniques or in a conventional argon-filled glovebox. Solvents were carefully refluxed over the appropriate drying agents and distilled prior to use: C_6_D_6_ and hexane (Na/K alloy), tetrahydrofuran (Na/benzophenone), and toluene (Na). Starting materials [Ta(η^5^-C_5_Me_5_)R(μ-S)]_2_ (R = Cl, Me, CH_2_Me, CH_2_SiMe_3_, C_3_H_5_, and Ph),^19^ and the organomagnesium reagents [MgR_2_(thf)_2_] (R = *p*-MeC_6_H_4_CH_2_, CH_2_Ph)^31^ were synthesized according to published procedures, and Li*n*Bu (1.6 M in hexane) was purchased by Sigma-Aldrich and used without further purification. Hydrogen was purchased from Linde. Microanalyses (C, H, N, and S) were performed on a LECO CHNS-932 microanalyzer. Samples for IR spectroscopy were prepared as KBr pellets and recorded on the PerkinElmer IR-FT Frontier or Bruker FT-IR-ALPHA II spectrophotometers (4000–400 cm^–1^). ^1^H and ^13^C NMR spectra were obtained by using Varian NMR System spectrometers: Unity-300 Plus, Mercury-VX, and Unity-500, and reported with reference to solvent resonances. ^1^H–^13^C gHSQC were recorded using the Unity-500 MHz NMR spectrometer operating at 25 °C.

#### Synthesis of [Ta(η^5^-C_5_Me_5_)*n*Bu(μ-S)]_2_ (**1**)

To a toluene solution (40 mL) of [Ta(η^5^-C_5_Me_5_)Cl(μ-S)]_2_ (0.500 g, 0.652 mmol) in a 100 mL Schlenk vessel was added a hexane solution of Li*n*Bu (0.8 mL, 1.303 mmol) at 0 °C. The reaction mixture was stirred at room temperature for 24 h and filtered through celite. The solvent was removed in vacuum to produce a green solid, which, after washing with hexane (3 × 10 mL), was identified as **1** (yield: 0.423 g, 80%). IR (KBr, cm^–1^) ν̅: 2955 (s, CH aliph.), 2912 (s, CH aliph.), 2866 (m, CH aliph.), 2850 (m, CH aliph.), 1484 (w, CC), 1427 (m, CC), 1377 (s, CC), 1027 (m, CC), 479 (w, Ta–C). ^1^H NMR (500 MHz, C_6_D_6_): δ 2.17 (s, 30H, C_5_*Me*_5_), 0.90–0.60 (m, 14H, CH_2_C*H*_2_C*H*_2_*Me*), −1.68 (m, 4H, C*H*_2_CH_2_CH_2_Me). ^13^C{^1^H} NMR (125 MHz, C_6_D_6_): δ 115.8 (*C*_5_Me_5_), 71.1 (*C*H_2_CH_2_CH_2_Me), 32.1, 30.3, 13.7 (CH_2_*C*H_2_*C*H_2_*Me*), 12.3 (C_5_*Me*_5_). Elemental Anal. (%) Calcd for C_28_H_48_S_2_Ta_2_ (810.71): C, 41.48; H, 5.97; S, 7.91. Found: C, 41.11; H, 5.47; S, 8.20.

#### Synthesis of [Ta(η^5^-C_5_Me_5_)CH_2_Ph(μ-S)]_2_ (**2**)

A 100 mL Schlenk vessel was charged in the glovebox with [Ta(η^5^-C_5_Me_5_)Cl(μ-S)]_2_ (0.500 g, 0.652 mmol), [Mg(CH_2_Ph)_2_(thf)_2_] (0.228 g, 0.652 mmol), and toluene (40 mL). After stirring for 24 h at room temperature, the reaction mixture was filtered through a medium-porosity glass frit, and the solvent was then removed in vacuum to yield **2** as a green solid after washing with hexane (3 × 10 mL) (yield: 0.515 g, 90%). IR (KBr, cm^–1^) ν̅: 3053 (w, CH arom.), 3017 (w, CH arom.), 2954 (w, CH aliph.), 2904 (m, CH aliph.), 2851 (w, CH aliph.), 1595 (m, CC), 1492 (s, CC), 1427 (m, CC), 1376 (s, CC), 1027 (s, CC), 483 (w, Ta–C). ^1^H NMR (500 MHz, C_6_D_6_): δ 7.05 (t, 4H, *J* = 10 Hz, H_m_, CH_2_*Ph*), 6.79 (t, 2H, *J* = 10 Hz, H_p_, CH_2_*Ph*), 6.68 (d, 4H, *J* = 10 Hz, H_o_, CH_2_*Ph*), 1.98 (s, 30H, C_5_*Me*_5_), −0.77 (s, 4H, C*H*_2_Ph). ^13^C{^1^H} NMR (125 MHz, C_6_D_6_): δ 143.4 (C_ipso_, CH_2_*Ph*), 130.2 (C_p_, CH_2_*Ph*), 127.7 (C_m_, CH_2_*Ph*), 123.1 (C_o_, CH_2_*Ph*), 116.6 (*C*_5_Me_5_), 76.5 (*C*H_2_Ph), 12.2 (C_5_*Me*_5_). Elemental Anal. (%) Calcd for C_34_H_44_S_2_Ta_2_ (878.74): C, 46.47; H, 5.05; S, 7.30. Found: C, 46.90; H, 4.98; S, 7.57.

#### Synthesis of [Ta(η^5^-C_5_Me_5_)(*p*-MeCH_2_Ph)(μ-S)]_2_ (**3**)

A 100 mL Schlenk vessel was charged in the glovebox with [Ta(η^5^-C_5_Me_5_)Cl(μ-S)]_2_ (0.500 g, 0.652 mmol), [Mg(*p*-MeC_6_H_4_CH_2_)(thf)_2_] (0.247 g, 0.652 mmol), and toluene (40 mL). After stirring for 24 h at room temperature, the reaction mixture was filtered through a medium-porosity glass frit, and the solvent was then removed in vacuum to yield **3** as a green solid after washing with hexane (3 × 10 mL) (yield: 0.562 g, 95%). IR (KBr, cm^–1^) ν̅: 3043 (w, CH arom.), 2974 (w, CH aliph.), 2904 (m, CH aliph.), 2852 (w, CH aliph.), 1634 (w, CC), 1608 (w, CC), 1506 (s, CC), 1453 (w, CC), 1428 (w, CC), 1378 (m, CC), 1028 (m, CC), 495 (w, Ta–C). ^1^H NMR (500 MHz, C_6_D_6_): δ 6.89 (t, 4H, *J* = 9 Hz, H_m_, *p*-MeC_6_*H*_4_CH_2_), 6.64 (d, 4H, *J* = 9 Hz, H_o_, *p*-MeC_6_*H*_4_CH_2_), 2.16 (s, 6H, *p*-*Me*C_6_H_4_CH_2_), 2.01 (s, 30H, C_5_*Me*_5_), −0.75 (s, 4H, *p*-MeC_6_H_4_C*H*_2_). ^13^C{^1^H} NMR (125 MHz, C_6_D_6_): δ 140.0 (C_ipso_, *p*-MeC_6_*H*_4_CH_2_), 132.1 (C_p_, *p*-Me*C*_6_H_4_CH_2_), 130.3 (C_m_, *p*-Me*C*_6_H_4_CH_2_), 128.4 (C_o_, *p*-Me*C*_6_H_4_CH_2_), 116.5 (*C*_5_Me_5_), 76.5 (*p*-MeC_6_H_4_*C*H_2_), 12.2 (C_5_*Me*_5_). Elemental Anal. (%) Calcd for C_34_H_44_S_2_Ta_2_ (906.79): C, 47.68; H, 5.33; S, 7.07. Found: C, 48.38; H, 4.90; S, 7.22.

#### Synthesis of [Ta(η^5^-C_5_Me_5_)(μ_3_-S)]_4_ (**4**)

A toluene solution (30–40 mL) of 0.500 g of [Ta(η^5^-C_5_Me_5_)R(μ-S)]_2_ (R = *n*Bu, 0.617 mmol; *p*-MeC_6_H_4_CH_2_, 0.551 mmol; CH_2_SiMe_3_, 0.574 mmol) was placed into a Carius tube (100 mL) with a Young’s valve. The argon pressure was reduced and replaced with hydrogen (*P* = 4.0 atm). The reaction mixture was stirred at room temperature for 24–48 h (or 70 °C, *P* < 1 atm, 24–48 h). The resulting solution was filtered, and the solvent was removed under vacuum to afford **4** as a dark green solid after washing with hexane (3 × 10 mL). (Yields: *n*Bu: 0.365 g, 85%; *p*-MeC_6_H_4_CH_2_: 0.346 g, 90%; CH_2_SiMe_3_: 0.380 g, 95%). IR (KBr, cm^–1^) ν̅: 2970 (m, CH aliph.), 2951 (m, CH aliph.), 2903 (s, CH aliph.), 1489 (w, CC), 1453 (m, CC), 1428 (m, CC), 1374 (s, CC), 1026 (s), 840 (m), 548 (m, Ta–C). ^1^H NMR (500 MHz, C_6_D_6_): δ 2.20 (s, 60 H, C_5_*Me*_5_). ^13^C{^1^H}NMR (125 MHz, C_6_D_6_): δ 107.2 (*C*_5_Me_5_), 12.8 (C_5_*Me*_5_). Elemental Anal. (%) Calcd for C_40_H_60_S_4_Ta_4_ (1392.96): C, 34.49; H, 4.34; S, 9.21. Found: C, 34.54; H, 4.57; S, 7.44. Repeated attempts to obtain satisfactory sulfur analysis for complex **4** were unsuccessful.

#### Synthesis of [Ta_2_(η^5^-C_5_Me_5_)_2_(H)Ph(μ-S)(μ_3_-S)]_2_ (**5**)

A toluene solution (20–30 mL) of [Ta(η^5^-C_5_Me_5_)Ph(μ-S)]_2_ (0.400 g, 0.470 mmol) was placed into a Fisher-Porter vessel (120 mL). The argon pressure was reduced and replaced with hydrogen pressure (*P* = 7 atm). The reaction mixture was left to heat at 60 °C for 4 days. The resulting solution was filtered, and the solvent was removed under vacuum to afford **5** as a dark orange solid (yield: 0.240 g, 66%). IR (KBr, cm^–1^) ν̅: 2978 (m, CH aliph.), 2903 (m, CH aliph.), 1633 (m, Ta–H), 1490 (w, CC), 1458 (w, CC), 1428 (w, CC), 1376 (m, CC), 1261 (w), 1027 (s), 882 (m), 731 (m), 700 (m). ^1^H NMR (500 MHz, C_6_D_6_): 7.10–6.70 (m, 10H, Ph), 2.20 (s, 30H, C_5_*Me*_5_), 1.87 (s, 30H, C_5_*Me*_5_), −4.98 (s, 2H, Ta–*H*). δ ^13^C{^1^H}NMR (125 MHz, C_6_D_6_): δ 138.6, 137.4, 125.5, 123.4 (Ph), 115.4, 112.3 (*C*_5_Me_5_), 13.4, 12.7 (C_5_*Me*_5_). Elemental Anal. (%) Calcd for C_52_H_72_S_4_Ta_4_·C_6_H_14_ (1635.36): C, 42.60; H, 5.30; S, 7.84. Found: C, 42.86; H, 4.77; S, 7.26.

#### Synthesis of [{Ta(η^5^-C_5_Me_5_)(η^3^-C_3_H_5_)}(μ-S)_2_{Ta(η^5^-C_5_Me_5_)(C_3_H_7_)}] (**6**)

A 120 mL Fisher-Porter vessel was charged in the glovebox with [Ta(η^5^-C_5_Me_5_)(η^3^-C_3_H_5_) (μ-S)]_2_ (0.400 g, 0.514 mmol) and 30–35 mL of toluene. The argon pressure was reduced and replaced with hydrogen pressure (*P* = 7 atm). The reaction mixture was left stirring at room temperature for 4 days. The resulting solution was filtered, and the solvent was removed under vacuum to afford **6** as a dark red solid after washing with hexane (3 × 10 mL), and the solvent was removed in vacuum (yield: 0.321 g, 80%). (KBr, cm^–1^) ν̅: 2974 (s, CH aliph.), 2944 (s, CH aliph.), 2907 (s, CH aliph.), 2858 (m, CH aliph.), 1493 (m, CC), 1375 (s, CC), 1214 (m, CC), 1027 (s, CC), 467 (m, Ta–C). ^1^H NMR (500 MHz, C_6_D_6_): 4.56 (A_4_X, 1H, ^3^*J* = 10 Hz, CH_2_C*H*CH_2_), 1.84, 1.78 (s, 15H, C_5_*Me*_5_), 1.49 (m, 2H, CH_2_C*H*_2_CH_3_) 0.84 (t, 3H, CH_2_CH_2_C*H*_3_), −0.85 (m, 2H, C*H*_2_CH_2_CH_3_), not observed (4H, C*H*_2_CHC*H*_2_). δ ^13^C{^1^H}NMR (125 MHz, C_6_D_6_): δ 114.7, 104.6 (*C*_5_Me_5_), 105.7 (CH_2_*C*HCH_2_), 66.8 (*C*H_2_CH*C*H_2_), 65.4 (*C*H_2_CH_2_CH_3_), 24.9 (CH_2_*C*H_2_CH_3_), 22.6 (CH_2_CH_2_*C*H_3_), 12.5, 11.8 (C_5_*Me*_5_). Elemental Anal. (%) Calcd for C_26_H_42_S_2_Ta_2_ (780.64): C, 40.00; H, 5.42; S, 8.21. Found: C, 39.72; H, 5.26; S, 8.10.

#### Synthesis of [Ta_2_(η^5^-C_5_Me_5_)_2_(μ-CH_2_C_6_H_6_)(μ-S)_2_] (**7**)

A toluene solution (40 mL) of [Ta(η^5^-C_5_Me_5_)(CH_2_Ph)(μ-S)]_2_ (0.500 g, 0.570 mmol) was placed into a Carius tube (150 mL) with a Young’s valve. The argon pressure was reduced and replaced with hydrogen pressure (*P* < 1 atm). The reaction mixture was heated to 65 °C for 48 h. The resulting solution was filtered, and the solvent was removed under vacuum to afford **6** as a dark yellow solid (yield: 0.310 g, 69%). IR (KBr, cm^–1^) ν̅: 3068 (w, CH arom.), 3023 (w, CH arom.), 2956 (m, CH aliph.), 2905 (s, CH aliph.), 2851 (w, CH aliph.), 1594 (w, CC), 1492 (m, CC), 1451 (m, CC), 1428 (s, CC), 1375 (s, CC), 1260 (m), 480 (w, Ta–C). ^1^H NMR (500 MHz, C_6_D_6_): 5.00, 3.71, 2.99, 2.27 (m, 4H, CH_2_C_6_*H*_6_), 3.44 (d, 1H, *J* = 10 Hz, CH_2_C_6_*H*_6_), 2.56 (dd, 1 H, *J* = 5 Hz; *J* = 11 Hz, CH_2_C_6_*H*_6_), 2.05 (s, 15H, C_5_*Me*_5_), 1.76 (s, 15H, C_5_*Me*_5_), 1.12 (AX, 1H, *J* = 10 Hz, C*H*_2_C_6_H_6_), 0.25 (AX, 1H, *J* = 10 Hz, C*H*_2_C_6_H_6_). δ ^13^C{^1^H}NMR (125 MHz, C_6_D_6_): δ 114.9, 107.2 (*C*_5_Me_5_), 114.2, 95.1, 81.9, 53.6 (CH_2_*C*_6_H_6_), 71.5 (C_ipso_, CH_2_*C*_6_H_6_), 65.7 (*C*H_2_C_6_H_6_), 29.3 (*C*H_2_*C*_6_H_6_), 12.4, 11.9 (C_5_*Me*_5_). Elemental Anal. (%) Calcd for C_27_H_38_S_2_Ta_2_ (788.62): C, 41.12; H, 4.86; S, 8.13. Found: C, 41.18; H, 4.72; S, 8.00.

### Crystal Structure Determination of Complexes **4–7**

Crystals were obtained by slow cooling at −20 °C of the corresponding toluene solutions. Single crystals were coated with mineral oil, mounted on Mitegen MicroMounts with the aid of a microscope, and immediately placed in the low-temperature nitrogen stream of the diffractometer. The intensity data sets for **4**, **5**, and **7** were collected at 200 K on a Bruker-Nonius KappaCCD diffractometer equipped with graphite-monochromated Mo Kα radiation (λ = 0.71073 Å) and an Oxford Cryostream 700 unit, while that for **6** was collected at 200 K on a Bruker D8 Venture diffractometer equipped with multilayer optics for monochromatization and collimator, Mo Kα radiation (λ = 0.71073 Å) and an Oxford Cryostream 800 unit. Crystallographic data for all complexes is presented in Table S1 in the Supporting Information.

The structures of compounds **4** and **7** were solved by direct methods (SHELXS),^32^ while those of **5** and **6** were solved by applying intrinsic phasing (SHELXT)^33^ using the WINGX^34^ or Olex2^35^ packages. All were refined by least-squares against *F*^2^ (SHELXL).^36^ All non-hydrogen atoms were anisotropically refined, while hydrogen atoms were placed at idealized positions and refined using a riding model, except the terminal hydride atom in complex **5**, which was localized in the difference Fourier map and isotropically refined, fixing both coordinates and thermal factor in the last cycles of refinement. Details about the absorption correction performed on each data set are described in the Supporting Information.

Complex **5** crystallized with one slightly disordered hexane solvent molecule; although it could be apparently modeled, a better refinement was achieved by using a solvent mask in Olex2^35^ removing the contribution of the disordered hexane molecule to the structure factors. Also, mild RIGU restraints were applied to the pentamethylcyclopentadienyl and phenyl carbon atoms.

Finally, two crystallographically independent molecules were found for compound **7**, where cyclopentadienyl carbon atoms C21–C25 and C41–C45 presented some dynamic disorder; thus, RIGU and SIMU restraints were applied.

### Computational Details

Calculations were performed using the Gaussian16 program package^37^ within the density functional theory (DFT)^38^ framework using the PBE0 functional.^39−41^ The geometries were obtained using a standard double-ξ Lanl2dz^42^ pseudopotential with an f polarization function^43^ to describe tantalum and a 6-31G(d,p) basis set^44−46^ for describing the rest of the atoms. To obtain the electronic energies, the basis set was extended to a triple-ξ pseudopotential Lanl2tz(f)^47^ for tantalum and to the augmented 6-311++G(2d,2p) basis set for the rest of the atoms.^48−50^ Toluene solvent effects were considered in all calculations with the IEF-PCM implicit solvation model^51^ as implemented in Gaussian16.^37^ We also applied Grimme’s GD3 dispersion correction.^52^ All the optimized minima were located without any restriction and in the absence of imaginary frequencies. Transition states were characterized by a single imaginary frequency, whose normal mode corresponded to the expected motion. In the calculation of Gibbs free energies, we used as a reference state 1 mol·L^–1^ in the condensed state, and frequencies below 100 cm^–1^ were withdrawn employing the Goodvibes code.^53^ The MECP between different spin states was located using the program developed by Harvey et al.^54^ using the EasyMECP code.^55^ A data set collection of the optimized structures for the most representative species is available in the ioChem-BD repository^56^ and can be accessed via https://iochem-bd.urv.es/browse/review-collection/100/1082/7d9fa995af391b9361a9b9de.

## Supporting Information Available

Cartesian coordinates for theoretical calculations (XYZ)
Crystallographic data for **4–7**; NMR and IR spectra for complexes **1–7**; frontier molecular orbital of complex **2**; and alternative pathways evaluated by DFT (PDF)

## Supplementary Material

ic3c00043_si_001.xyz
