# Supplementary material for: Reductive Hydrogenation of Sulfido-Bridged Tantalum Alkyl Complexes: A Mechanistic Insight
Source: Inorg Chem. 2023 Jun 15;62(26):10100–9. doi: 10.1021/acs.inorgchem.3c00043 (PMC10324320; doi:10.1021/acs.inorgchem.3c00043)
Supplement: Supplementary file 2 — ic3c00043_si_002.pdf [file ic3c00043_si_002.pdf]

Supporting Information for the Paper Entitled

Reductive Hydrogenation of Sulfido-Bridged Tantalum Alkyl Complexes: A Mechanistic Insight

Jorge J. Carbó,[b,]* *Manuel Gómez,*[a] *Cristina Hernández-Prieto,*[a] *Alberto Hernán-Gómez,*[a] *Avelino Martín,*[a] *Miguel Mena,*[a] *Jordi Puiggalí-Jou,* [b] *Josep M. Ricart,*[b] *and Cristina Santamaría,*[a,*]

[a] Departamento de Química Orgánica y Química Inorgánica, Instituto de Investigación Química "Andrés M. del Río" (IQAR), Universidad de Alcalá, Campus Universitario, E-28805 Alcalá de Henares, Madrid, Spain. [b] Departament de Química Física i Inorgànica, Universitat Rovira i Virgili, C/ Marcel.lí Domingo, s/n, 43007 Tarragona, Spain.

Table of Contents

Crystallographic Data

Table S1. Experimental data for the X-ray diffraction studies on **4-7** S3

Absorption correction procedures for **4-7** S4

Spectroscopic Characterization

NMR and IR Spectra for complexes **1**-**7** (**Figures S1-S26**) S5-S18

Computational study S19-S20

Crystallographic Data

Table S1. Experimental data for the X-ray diffraction studies on complexes **4**, **5**, **6**, and **7**.

	**4**	**5**	**6**	**7**
Formula	$C_{40}H_{60}S_4Ta_4$	$C_{52}H_{72}S_4Ta_4$	$C_{26}H_{42}S_2Ta_2$	$C_{27}H_{38}S_2Ta_2$
M	1392.92	1549.13	780.61	788.59
T [K]	200(2)	200(2)	200(2)	200(2)
λ[Å]	0.71073	0.71073	0.71073	0.71073
Crystal system	Tetragonal	monoclinic	triclinic	triclinic
Space group	*I*-4	*C*2/c	*P* -1	*P* -1
a [Å]; α [º]	12.1481(7)	18.043(2)	9.2359(9); 78.902(3)	8.9050(6); 74.340(6)
b [Å]; β [º]	12.1481(7)	12.738(1); 96.16(1)	10.838(1); 80.070(3)	15.150(1); 89.276(6)
c [Å]; γ [º]	14.4068(3)	24.848(2)	15.069(2); 66.303(3)	19.477(2); 88.327(6)
V [$Å^3$]	2126.1(2)	5678(1)	1347.8(2)	2528.9(3)
Z	2	4	2	4
ρ_{calcd} [g cm^{-3}]	2.176	1.812	1.924	2.071
μ [mm^{-1}]	10.398	7.860	8.279	8.826
F(000)	1312	2960	752	1512
Crystal size [mm^3]	0.26 x 0.20 x 0.19	0.25 x 0.18 x 0.16	0.28 x 0.17 x 0.14	0.31 x 0.17 x 0.11
θ range [deg]	3.35 to 25.02	2.27 to 25.02	3.23 to 27.53	3.01 to 25.03
Index ranges	-14 to 14, -14 to 14, -16 to 17	21 to -21, 14 to -15, 29 to -29	11 to -11, 14 to -12, 19 to -19	-10 to 10, -17 to 17, -23 to 23
Reflections collected	10063	35491	32747	40274
Unique data	1887 (R_{int} = 0.098)	5004 (R_{int} = 0.099)	6154 (R_{int} = 0.045)	8822 (R_{int} = 0.126)
Reflections [I>2σ(I)]	1732	3306	5096	6317
Goodness-of-fit on F^2	1.134	1.060	1.052	1.08
Final R indices [I>2σ(I)]	R1 = 0.035, wR2 = 0.079	R1 = 0.049; wR2 = 0.104	R1 = 0.025; wR2 = 0.058	R1 = 0.046; wR2 = 0.102
R indices (all data)	R1 = 0.044 wR2 = 0.084	R1 = 0.104; wR2 = 0.133	R1 = 0.034, wR2 = 0.061	R1 = 0.086, wR2 = 0.128
Largest diff. peak/hole [e·$Å^{-3}$]	1.893 / -1.113	2.125 / -1.366	2.841 / -1.288	2.796 / -2.388

[a] $R1 = \Sigma||F_o|-|F_c||/[\Sigma|F_o|]$ $wR2 = \{[\Sigma w(F_0^2 - F_c^2)^2] / [\Sigma w(F_0^2)^2]\}^{1/2}$

Absorption correction procedures for 4-7

The composition of complexes **4-7** together with the poor quality of the crystals, in some cases, difficulted to obtain crystal structure determinations with low residual peaks. Complexes **4-7** presented important absorption coefficients (see **Table S1**) due to the presence of tantalum and sulfur atoms, hence several absorption correction methods have been tried to get good refinements.

In the case of complex **4** SORTAV[1] multi-scan absorption correction was used in first place (2.152/-0.897 e·Å^{-3}), but ANALYTICAL[2] methods were finally applied to reduce the number and magnitude of the residuals (1.893 / -1.113 e·Å^{-3}).

Numerical SADABS[3] absorption correction was performed for complex **5** (2.125 / -1.366 e·Å^{-3}). Other methods like multi-scan SORTAV[1] (2.110/-2.501 e·Å^{-3}) or SADABS[3] (2.710/ -1.93 e·Å^{-3}) were also used to improve the quality of the absorption correction but obtained worse results.

Multi-scan SADABS[3] gave the best results for the absorption correction of complex **6** data set, while for **7** was SORTAV[1] (2.796 /-2.388 e·Å^{-3}), giving multi-scan SADABS[3] (3.23/-2.88 e·Å^{-3}) or ANALYTICAL[2] (3.83/ -4.97 e·Å^{-3}) clearly higher peaks.

---

[1] Blessing, R. H. An empirical correction for absorption anisotropy. *Acta Cryst.* **1995**, *A51*, 33-38.
[2] Alcock, N. W. *Crystallographic Computing*, **1970**, edited by F. R. Ahmed, S.R. Hall & C. P. Huber, p. 271. Copenhagen: Munksgaard
[3] Krause, L.; Herbst-Irmer, R.; Sheldrick, G.M.; Stalke, D. Comparison of silver and molybdenum microfocus X-ray sources for single-crystal structure determination. *J. Appl. Cryst.* **2015**, *48*, 3-10.

NMR Spectroscopy

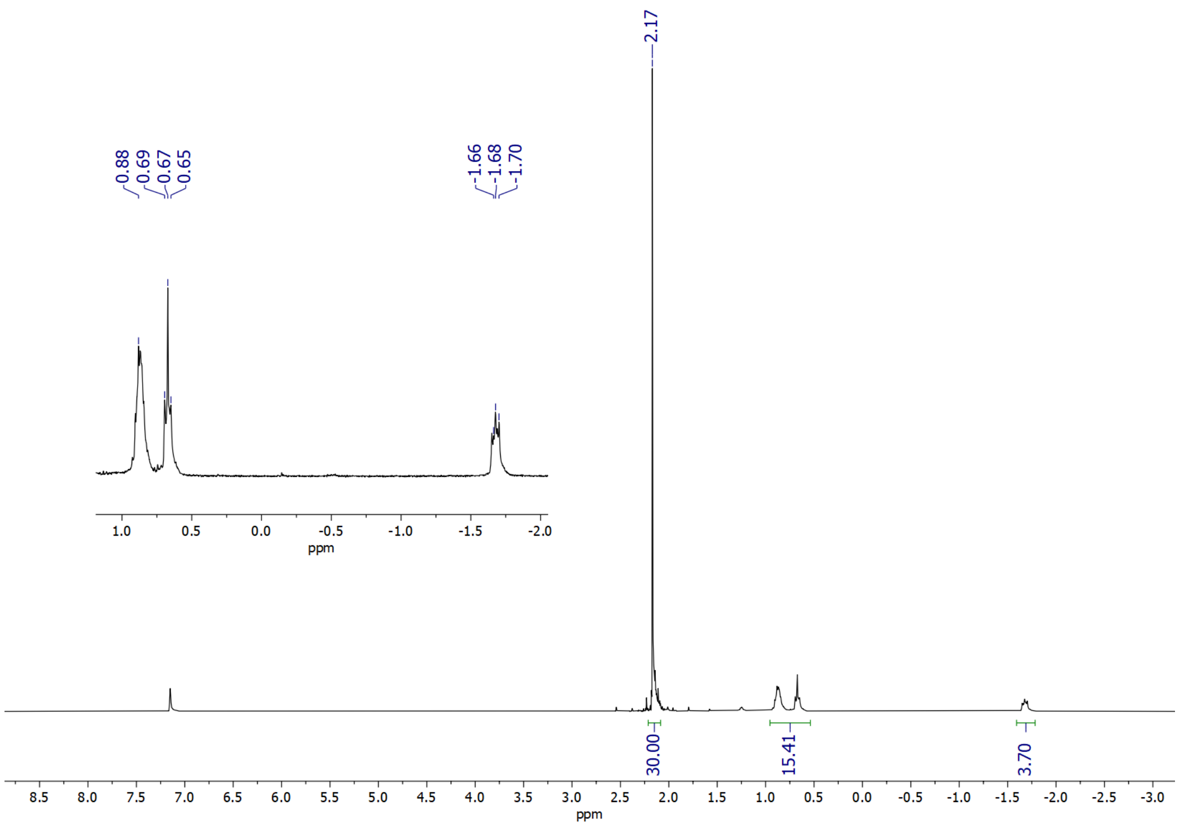

Figure S1. ^{1}H NMR spectrum of compound $[Ta(\eta^5\text{-}C_5Me_5)n\text{Bu}(\mu\text{-S})]_2$ (**1**) in C_6D_6 (500 MHz).

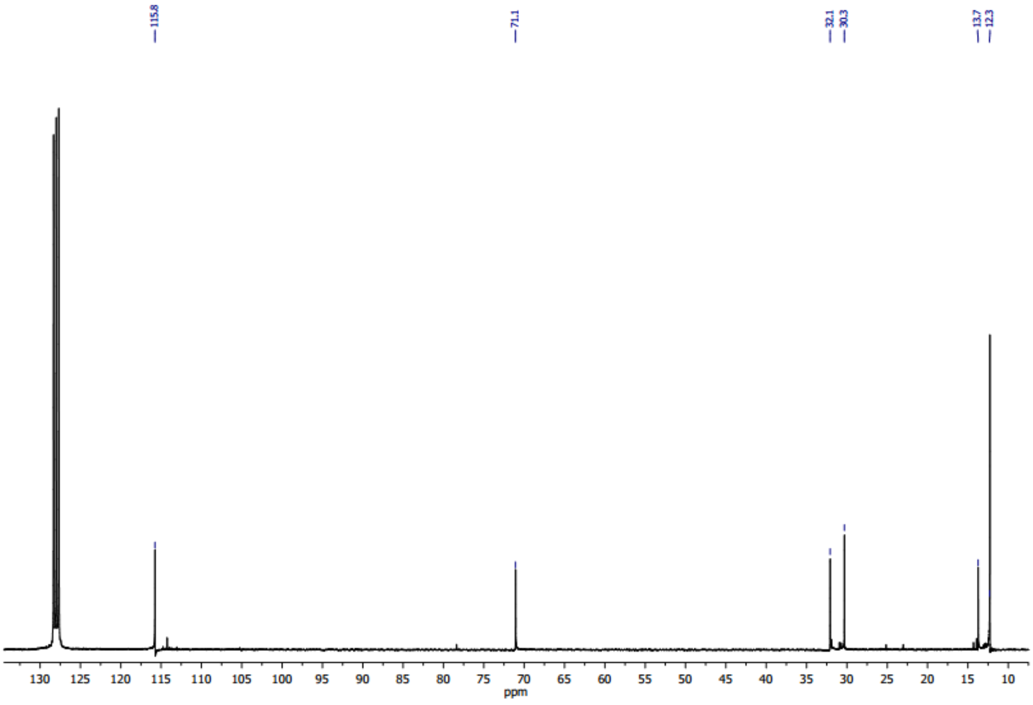

Figure S2. ^{13}C NMR spectrum of compound $[Ta(\eta^5\text{-}C_5Me_5)n\text{Bu}(\mu\text{-S})]_2$ (**1**) in C_6D_6 (125 MHz).

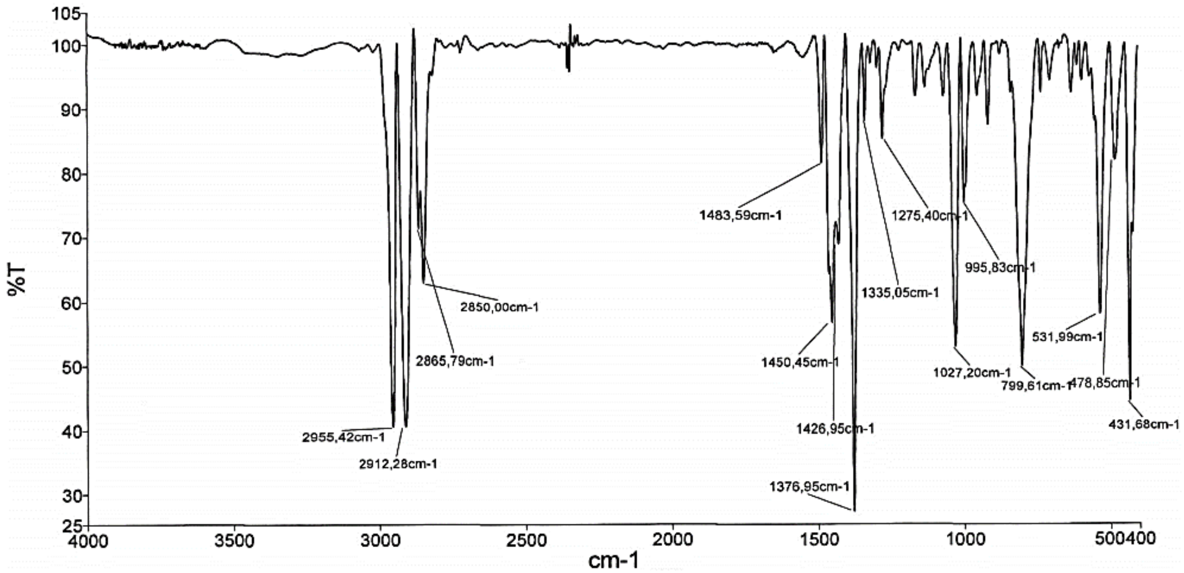

Figure S3. IR spectrum (KBr) for complex [Ta(η^5-C_5Me_5)*n*Bu(μ-S)]$_2$ (**1**).

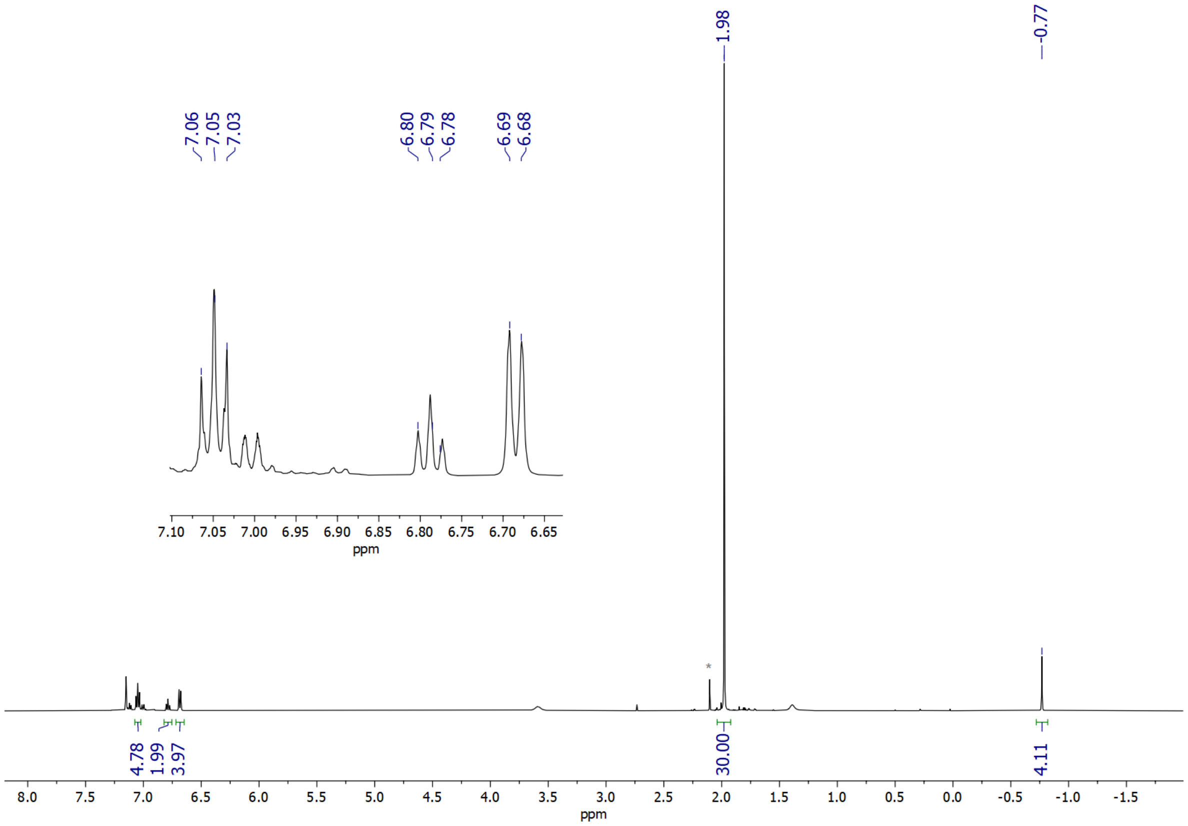

Figure S4. ^{1}H NMR spectrum of compound [Ta(η^5-C_5Me_5)(CH_2Ph)(μ-S)]$_2$. (**2**) in C_6D_6 (500 MHz) (*toluene).

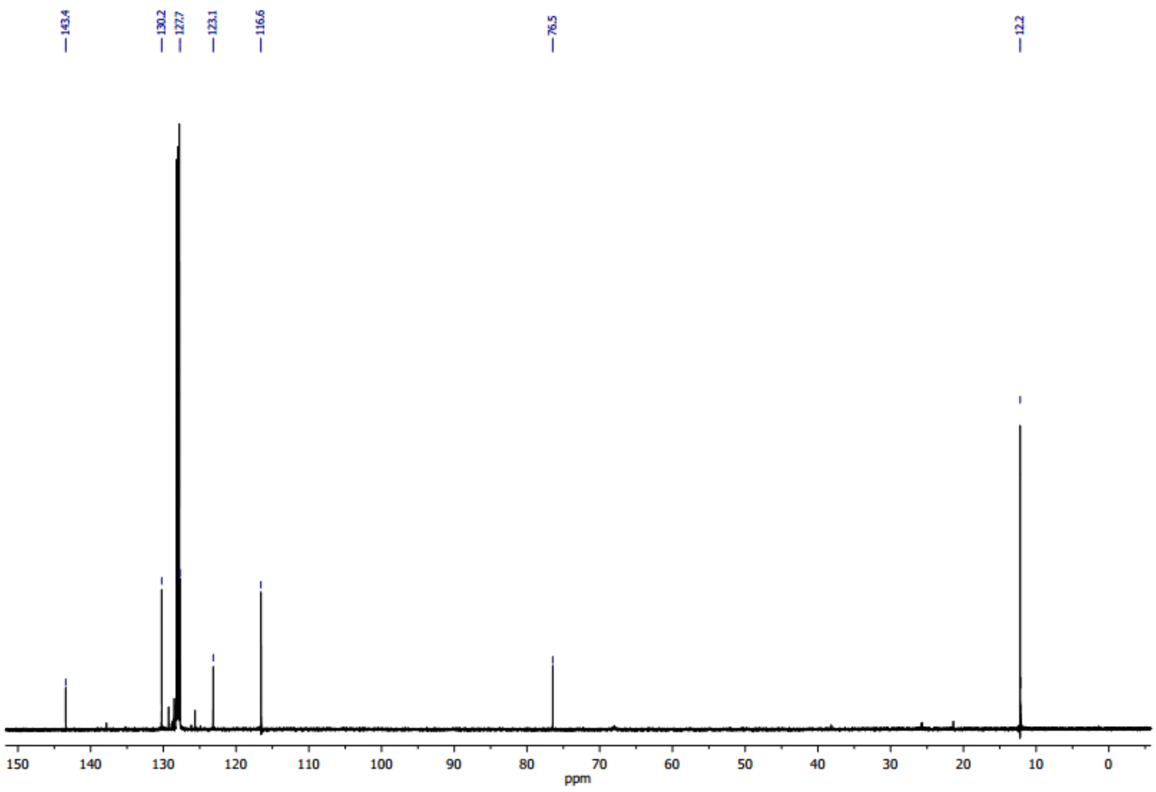

Figure S5. ^{13}C NMR spectrum of compound [Ta(η^5-C_5Me_5)(CH_2Ph)(μ-S)]$_2$ (**2**) in C_6D_6 (125 MHz).

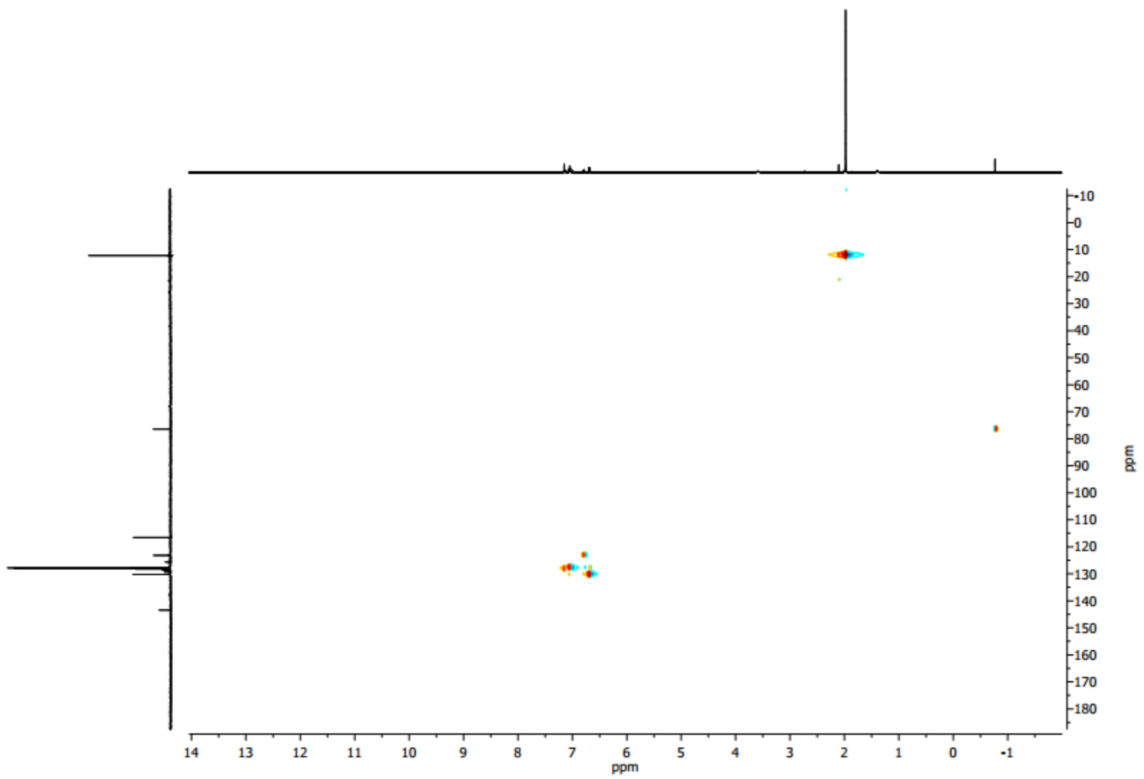

Figure S6. g-HSQC NMR spectrum of compound [Ta(η^5-C_5Me_5)(CH_2Ph)(μ-S)]$_2$ (**2**) in C_6D_6 (500 MHz).

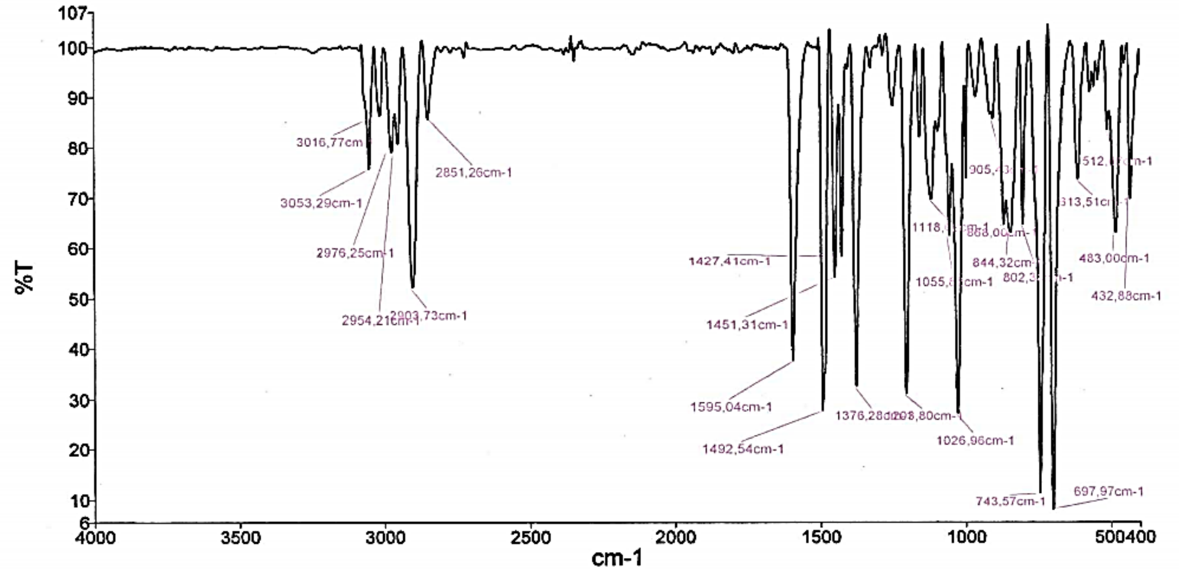

Figure S7. IR spectrum (KBr) for complex [Ta(η^5-C_5Me_5)(CH_2Ph)(μ-S)]$_2$ (**2**).

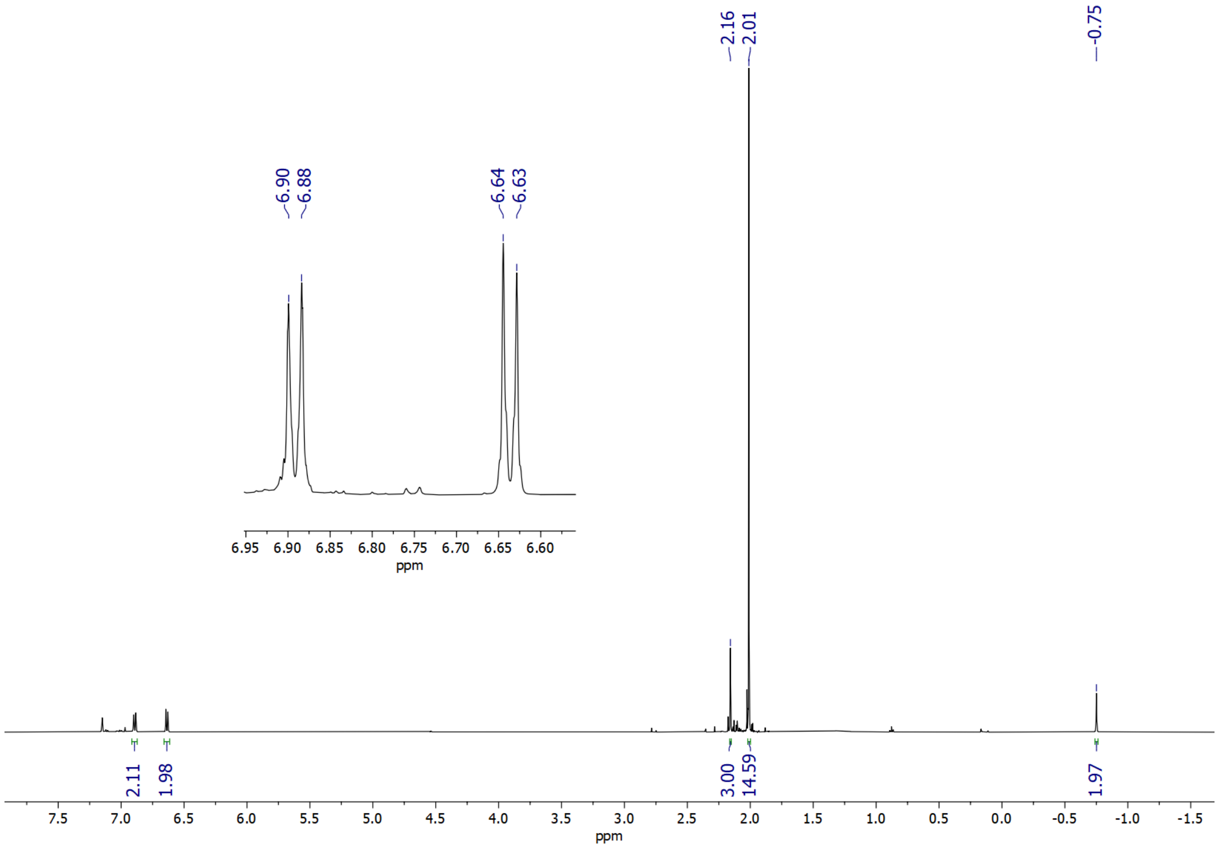

Figure S8. ^{1}H NMR spectrum of compound [Ta(η^5-C_5Me_5)(*p*-$MeC_6H_4CH_2$)(μ-S)]$_2$ (**3**) in C_6D_6 (500 MHz).

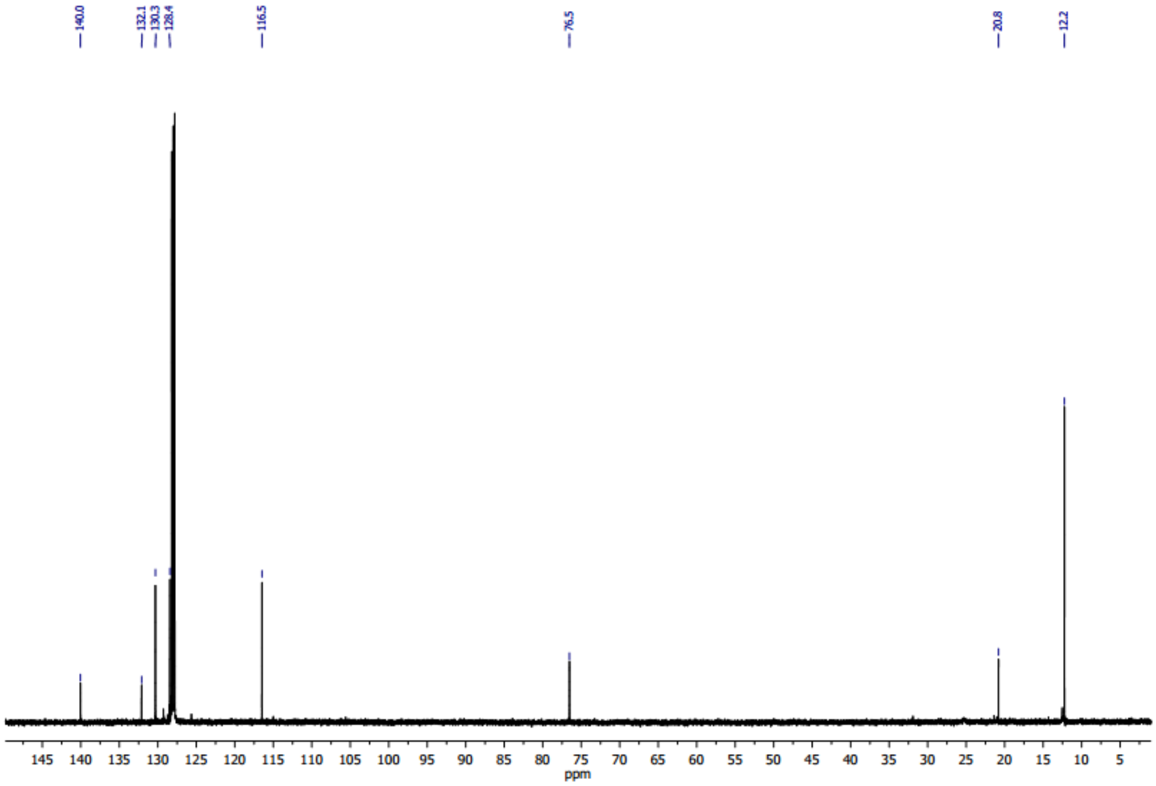

Figure S9. ^{13}C NMR spectrum of compound [Ta(η^5-C_5Me_5)(p-$MeC_6H_4CH_2$)(μ-S)]$_2$ (**3**) in C_6D_6 (125 MHz).

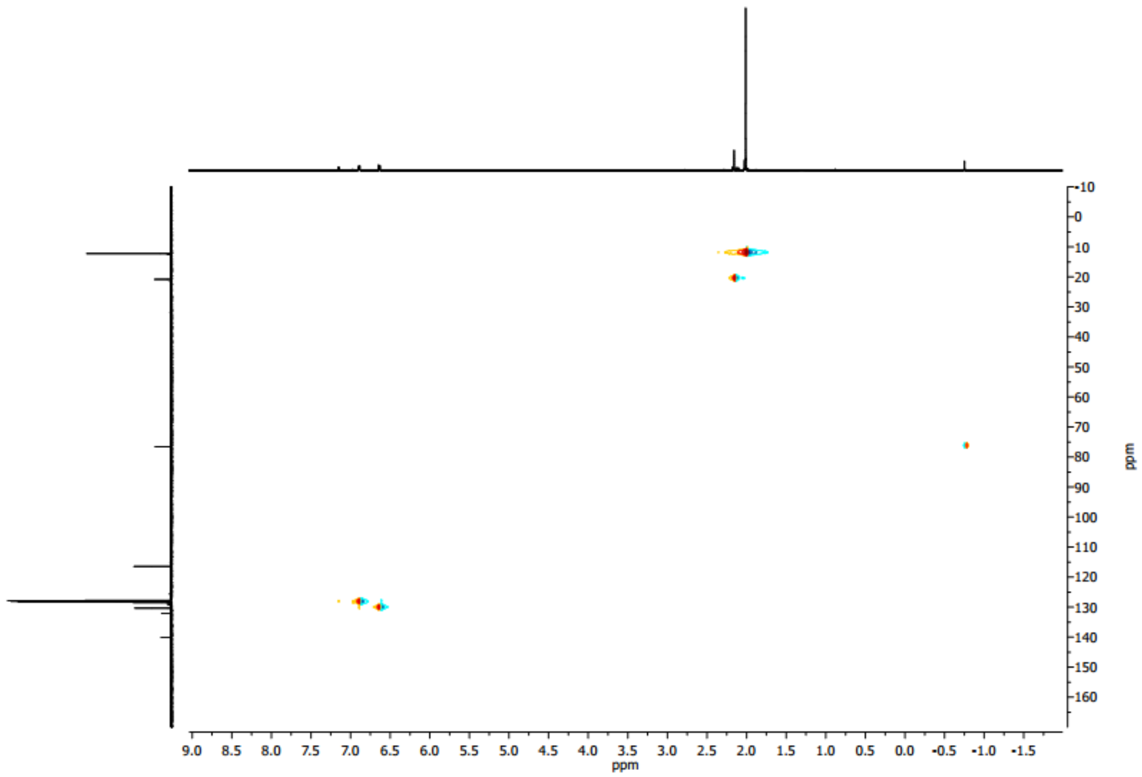

Figure S10. g-HSQC NMR spectrum of compound [Ta(η^5-C_5Me_5)(p-$MeC_6H_4CH_2$)(μ-S)]$_2$ (**3**) in C_6D_6 (500 MHz).

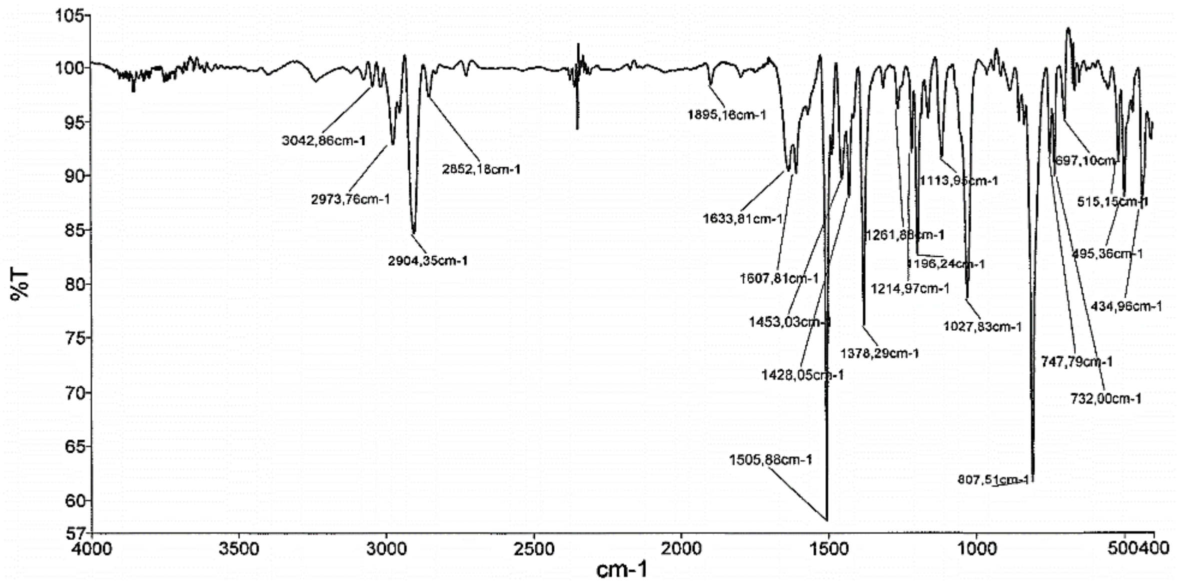

Figure 11. IR spectrum (KBr) for complex [Ta(η^5-C_5Me_5)(*p*-$MeC_6H_4CH_2$)(μ-S)]$_2$ (**3**).

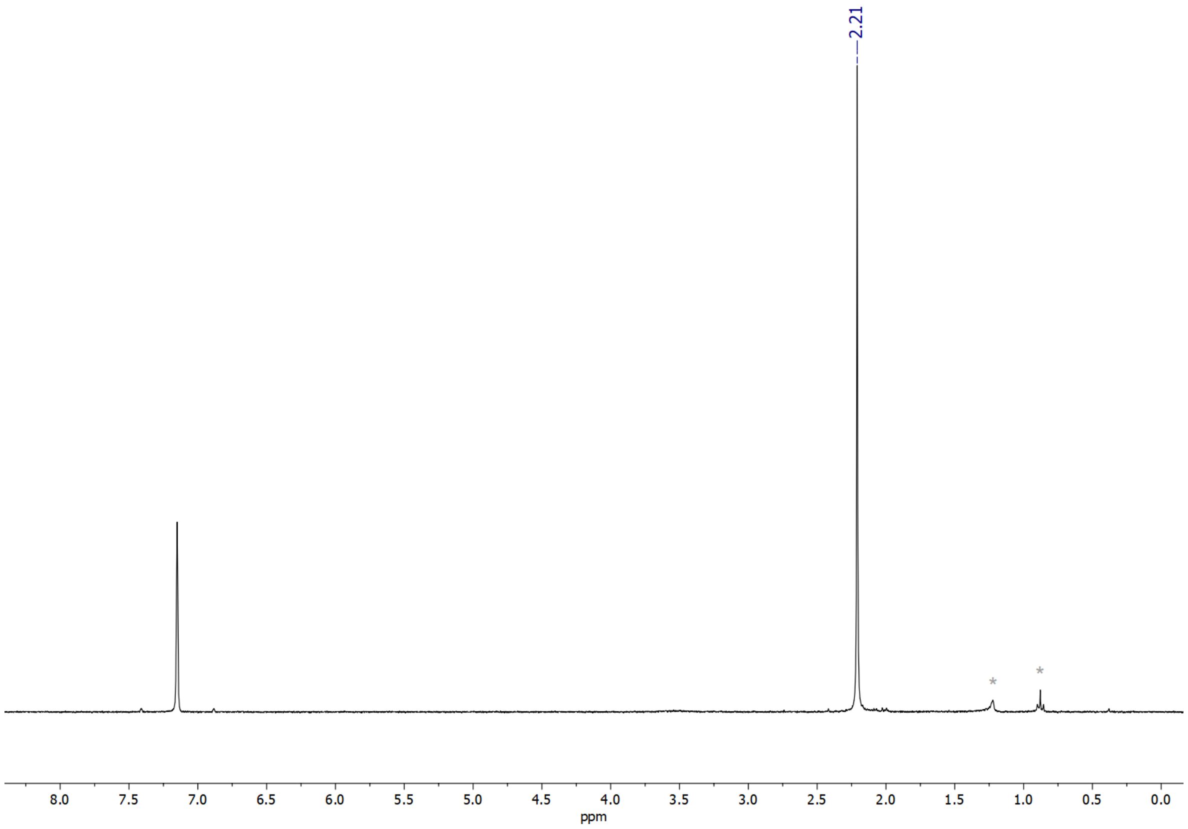

Figure S12. ^{1}H NMR spectrum of compound [Ta(η^5-C_5Me_5)(μ_3-S)]$_4$ (**4**) in C_6D_6 (500 MHz) (*hexane).

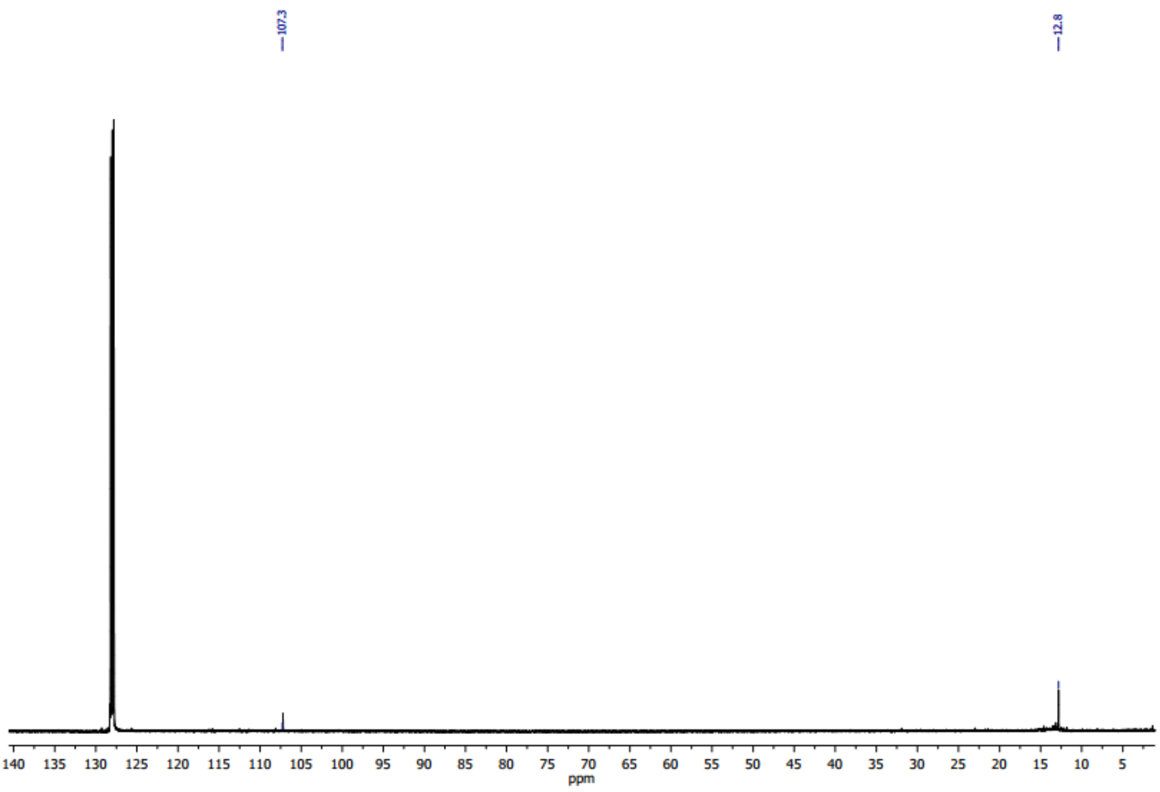

Figure S13. ^{13}C NMR spectrum of compound [Ta(η^5-C_5Me_5)(μ_3-S)]$_4$ (**4**) in C_6D_6 (125 MHz).

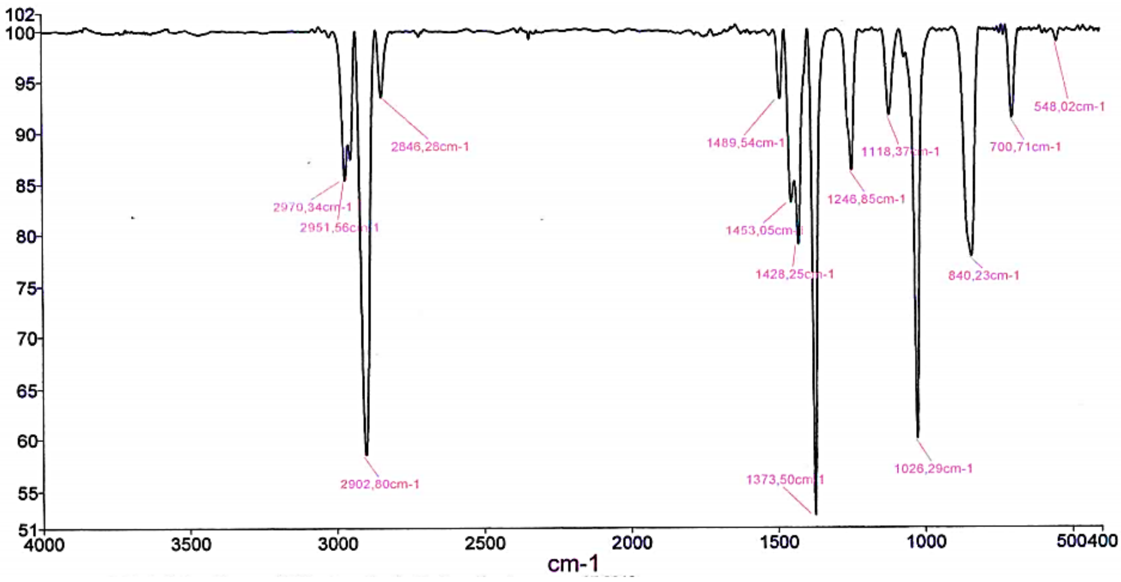

Figure 14. IR spectrum (KBr) for complex [Ta(η^5-C_5Me_5)(μ_3-S)]$_4$ (**4**).

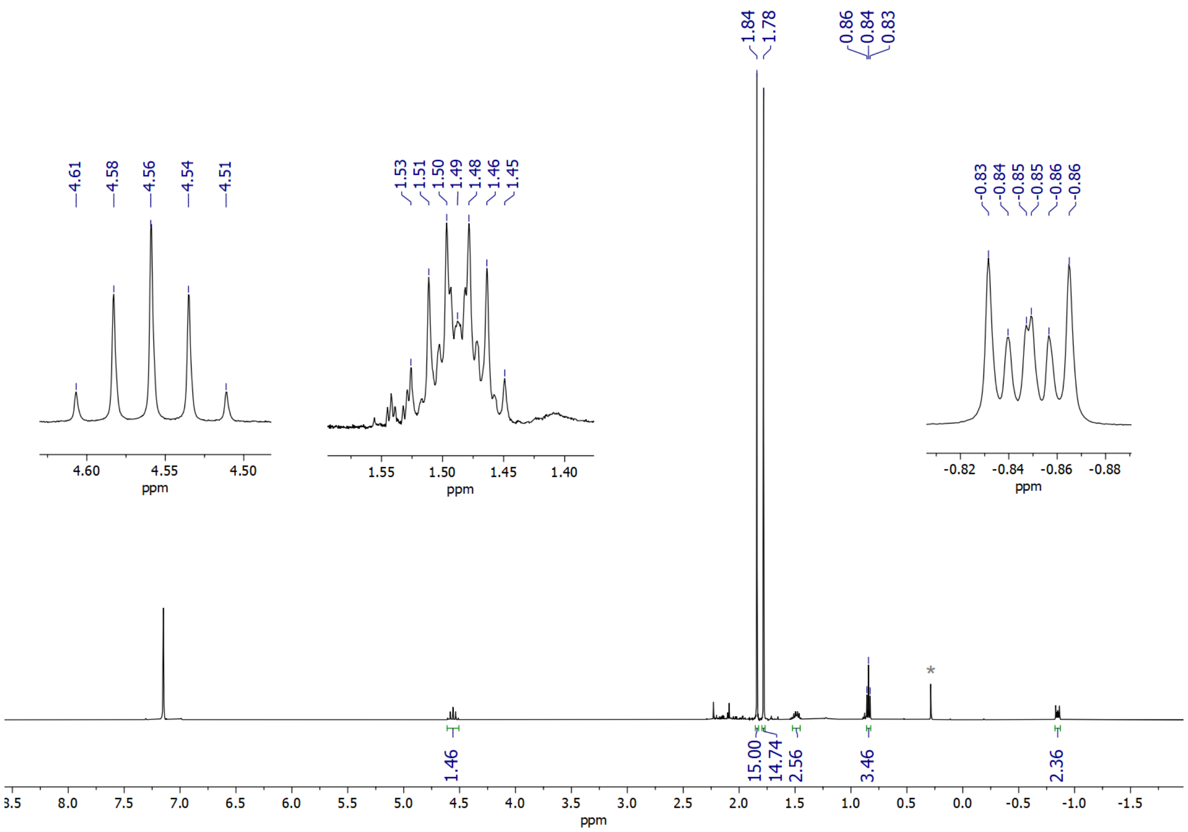

Figure S15. ^{1}H NMR spectrum of compound [{Ta(η^5-C_5Me_5)(η^3-C_3H_5)}(μ-S){Ta(η^5-C_5Me_5)(C_3H_7)}] (**5**) in C_6D_6 (500 MHz) (*grease).

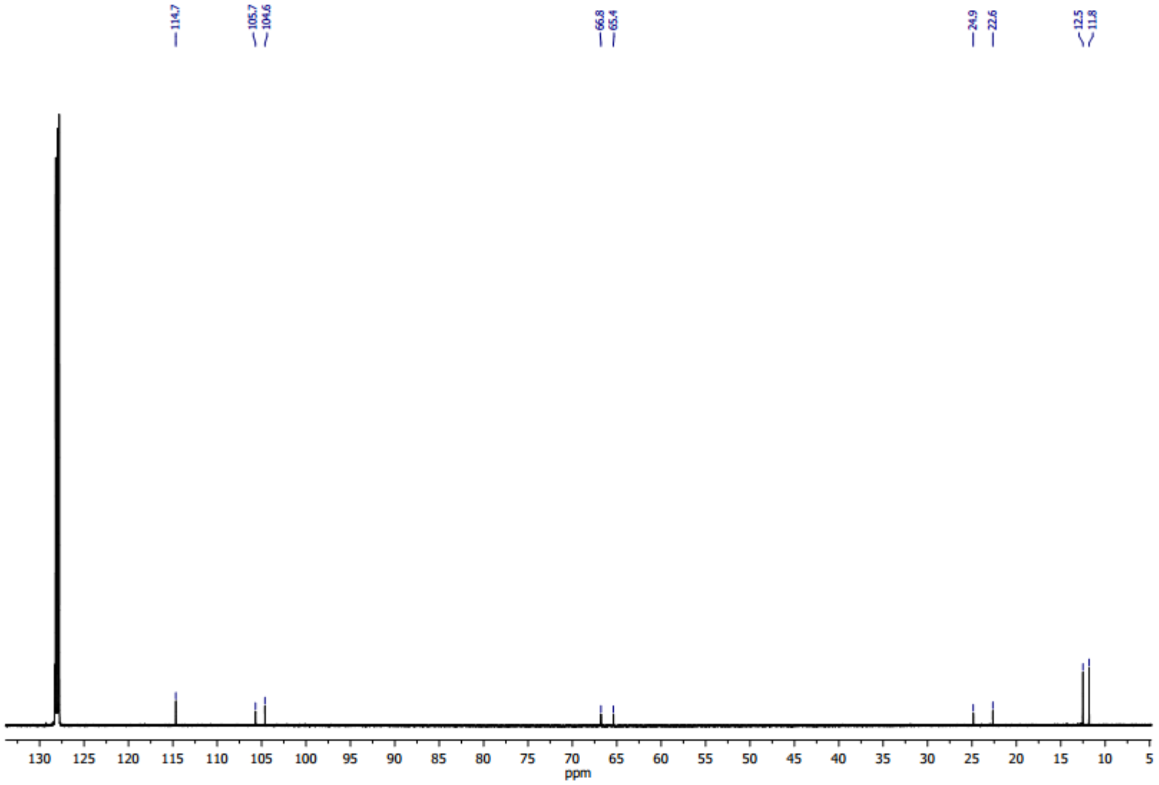

Figure S16. $^{13}C\{^{1}H\}$ NMR spectrum of compound [{Ta(η^5-C_5Me_5)(η^3-C_3H_5)}(μ-S){Ta(η^5-C_5Me_5)(C_3H_7)}] (**5**) in C_6D_6 (125 MHz).

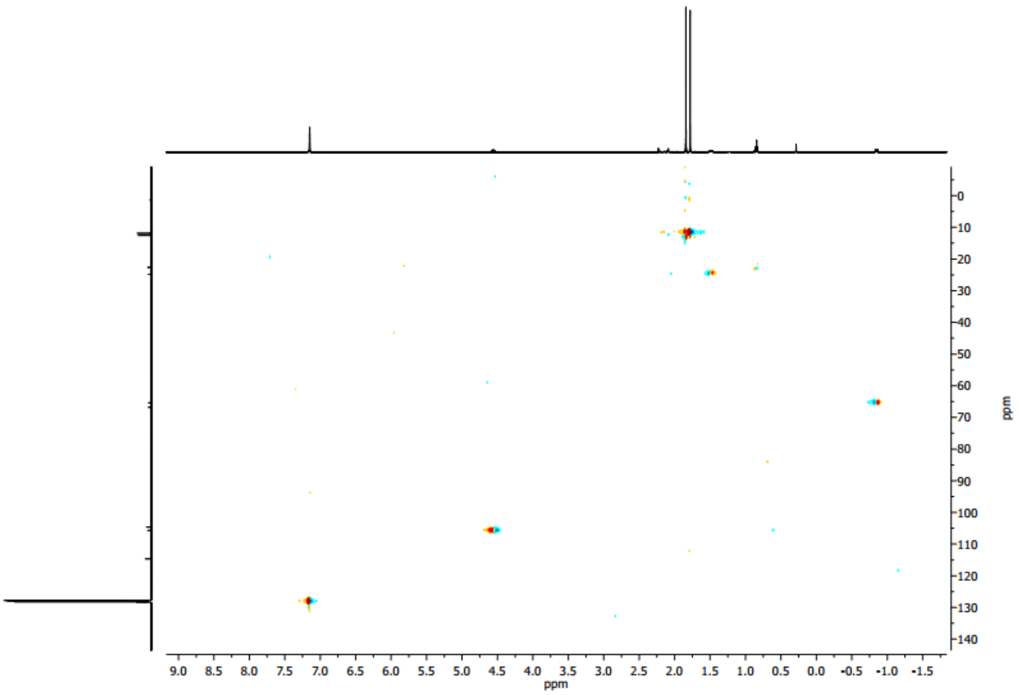

Figure S17. g-HSQC NMR spectrum of compound [{Ta(η^5-C_5Me_5)(η^3-C_3H_5)}(μ-S){Ta(η^5-C_5Me_5)(C_3H_7)}] (**5**) in C_6D_6 (500 MHz).

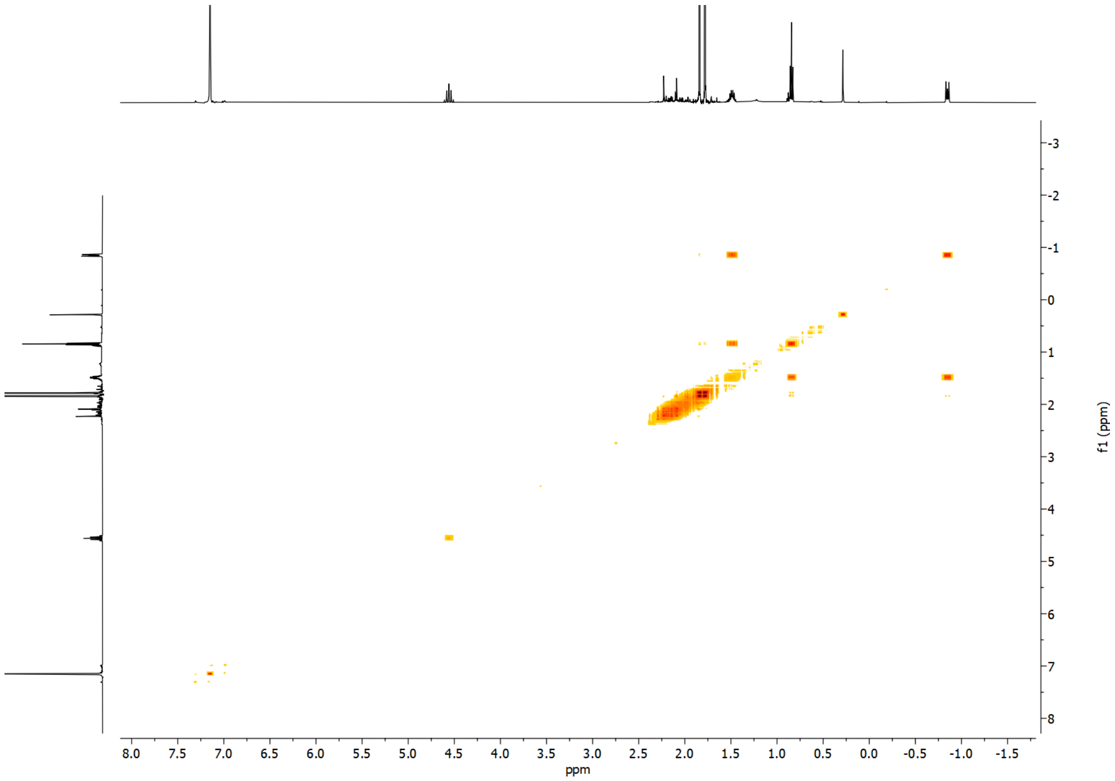

Figure S18. HOMO-COSY spectrum of compound [{Ta(η^5-C_5Me_5)(η^3-C_3H_5)}(μ-S){Ta(η^5-C_5Me_5)(C_3H_7)}] (**5**) in C_6D_6 (500 MHz).

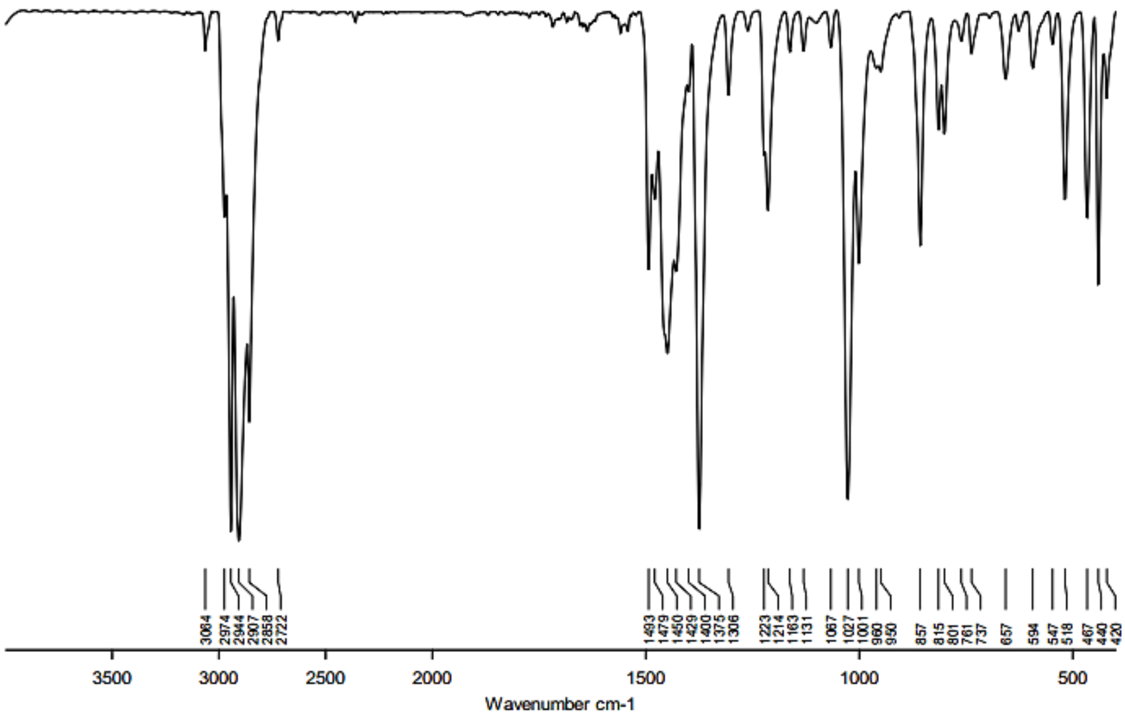

Figure S19. IR spectrum (KBr) for complex [{Ta(η^5-C_5Me_5)(η^3-C_3H_5)}(μ-S){Ta(η^5-C_5Me_5)(C_3H_7)}] (**5**).

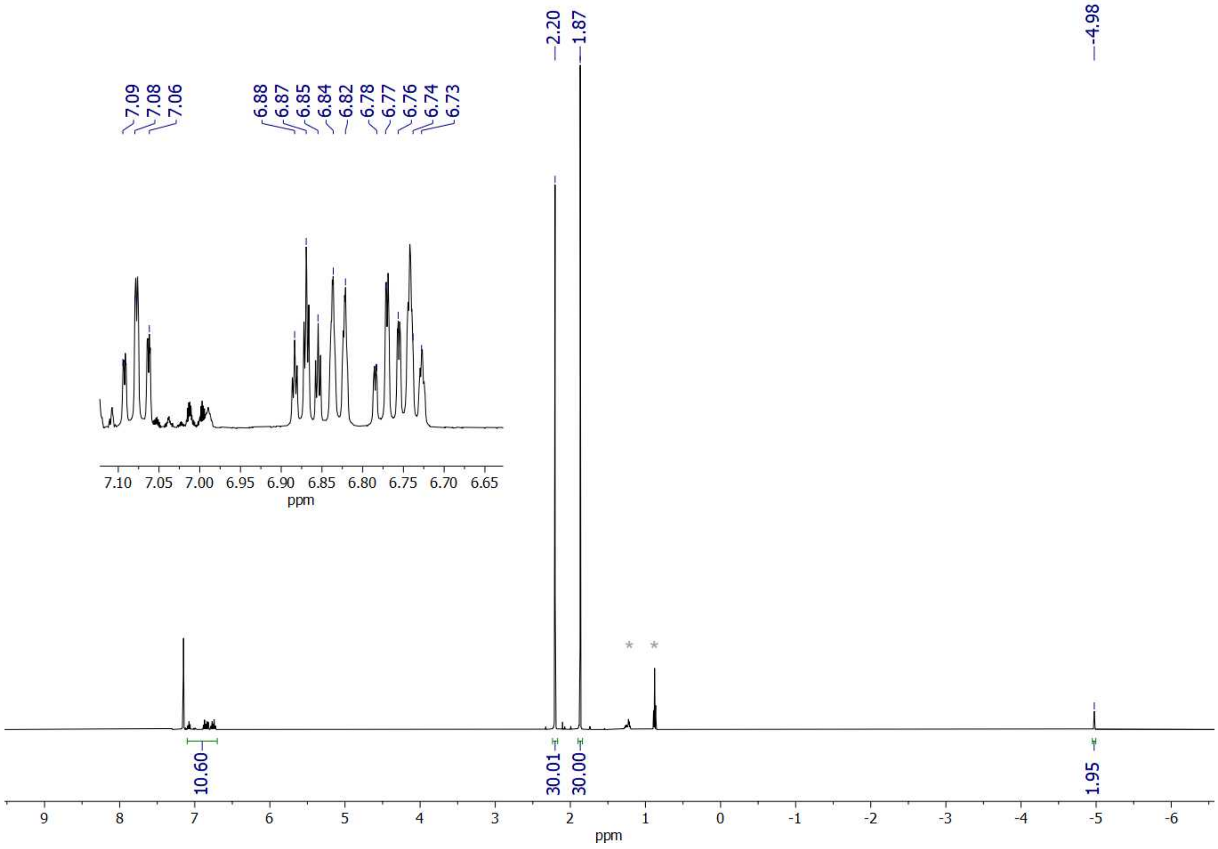

Figure S20. ^{1}H NMR spectrum of compound [Ta_2(η^5-C_5Me_5)$_2$(H)Ph(μ-S)(μ_3-S)]$_2$ (**6**) in C_6D_6 (500 MHz) (*hexane).

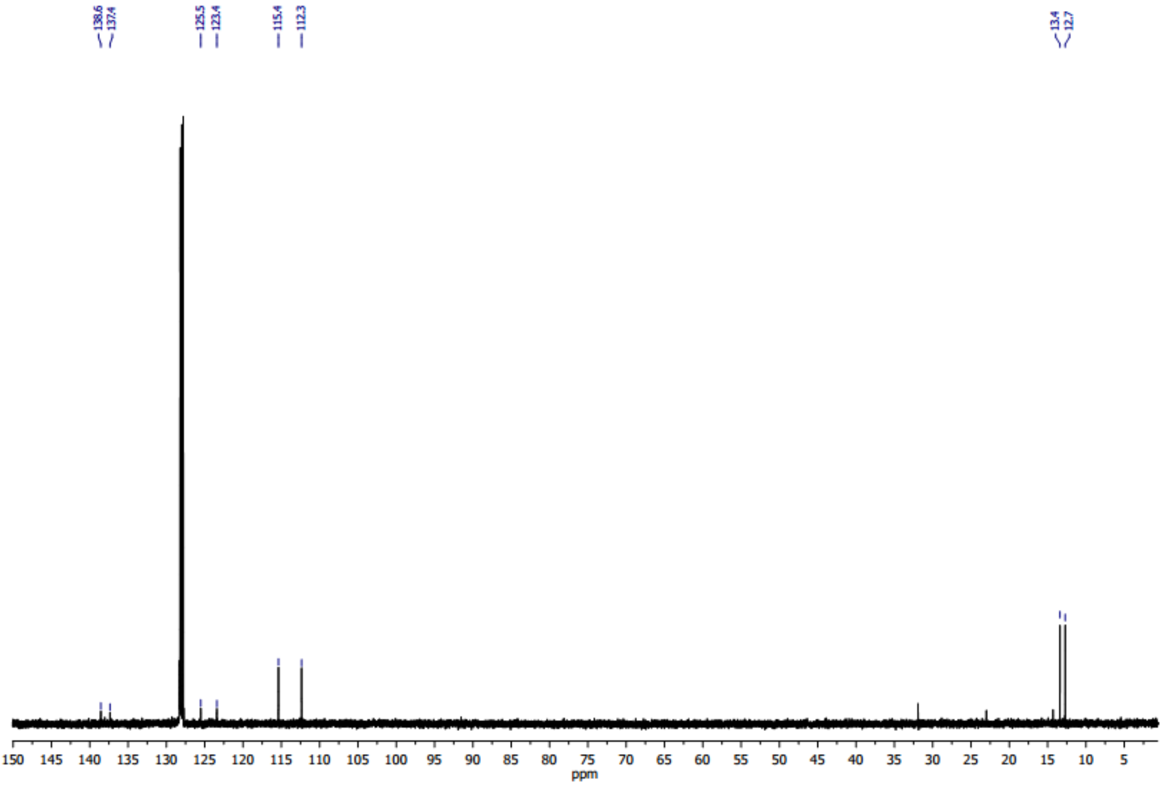

Figure S21. $^{13}C\{^1H\}$ NMR spectrum of compound $[Ta_2(\eta^5\text{-}C_5Me_5)_2(H)Ph(\mu\text{-}S)(\mu_3\text{-}S)]_2$ (**6**) in C_6D_6 (125 MHz).

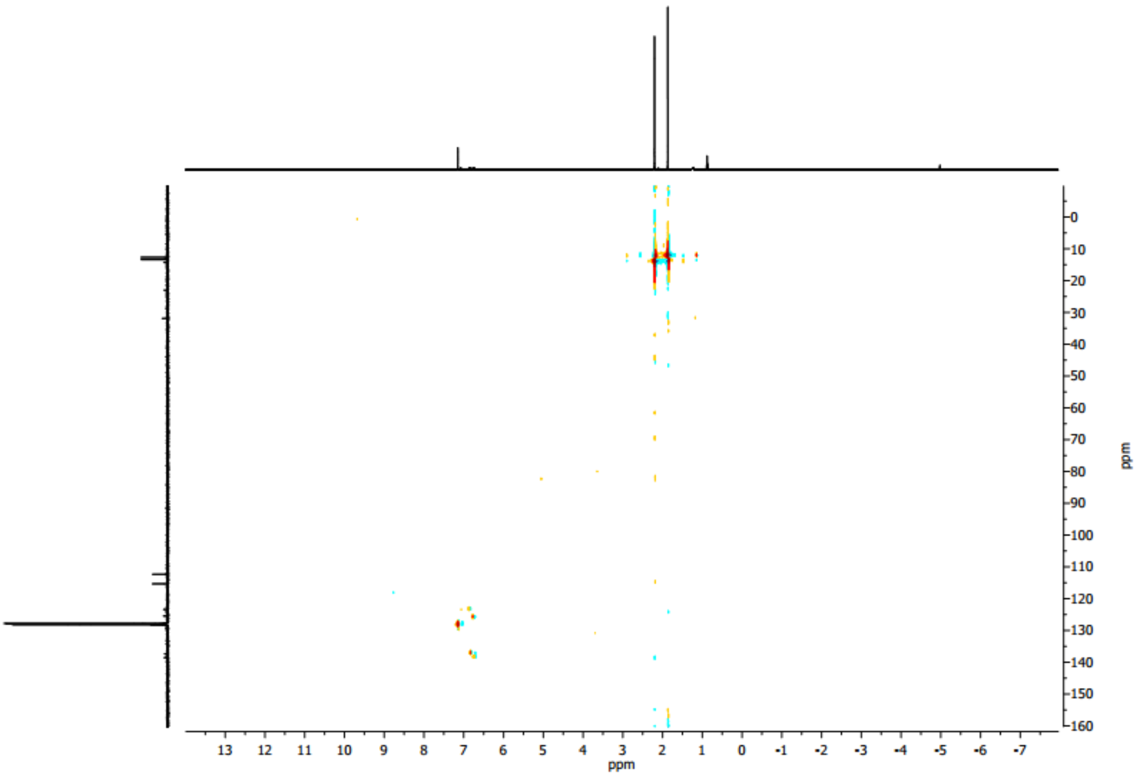

Figure S22. g-HSQC NMR spectrum of compound [$Ta_2(\eta^5\text{-}C_5Me_5)_2(H)Ph(\mu\text{-}S)(\mu_3\text{-}S)]_2$ (**6**) in C_6D_6 (500 MHz).

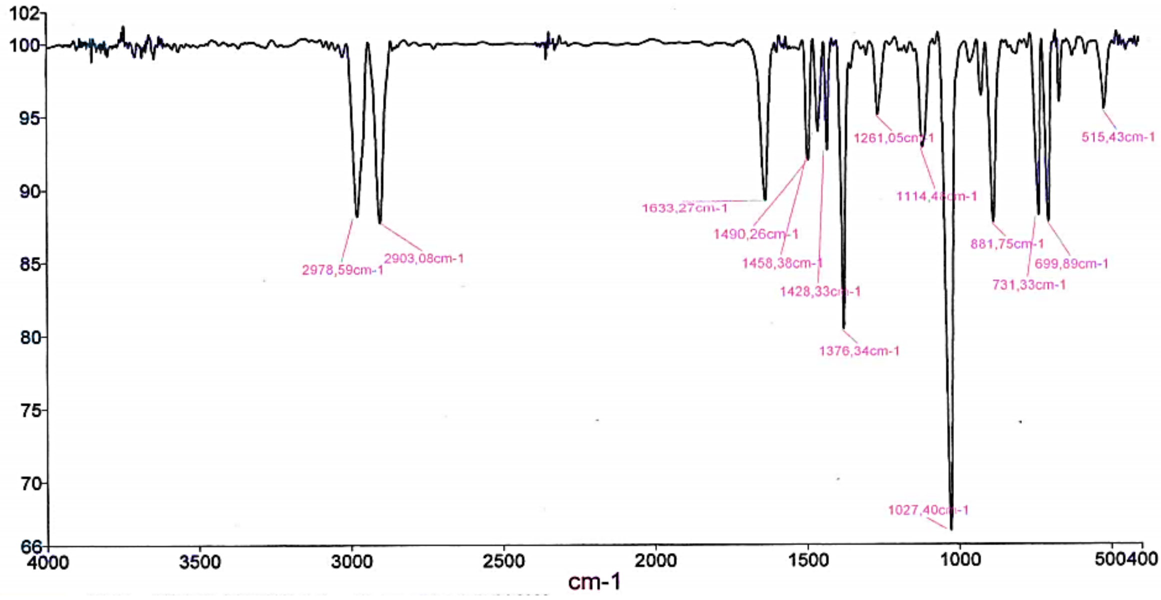

Figure S23. IR spectrum (KBr) for complex [$Ta_2(\eta^5\text{-}C_5Me_5)_2(H)Ph(\mu\text{-}S)(\mu_3\text{-}S)]_2$ (**6**).

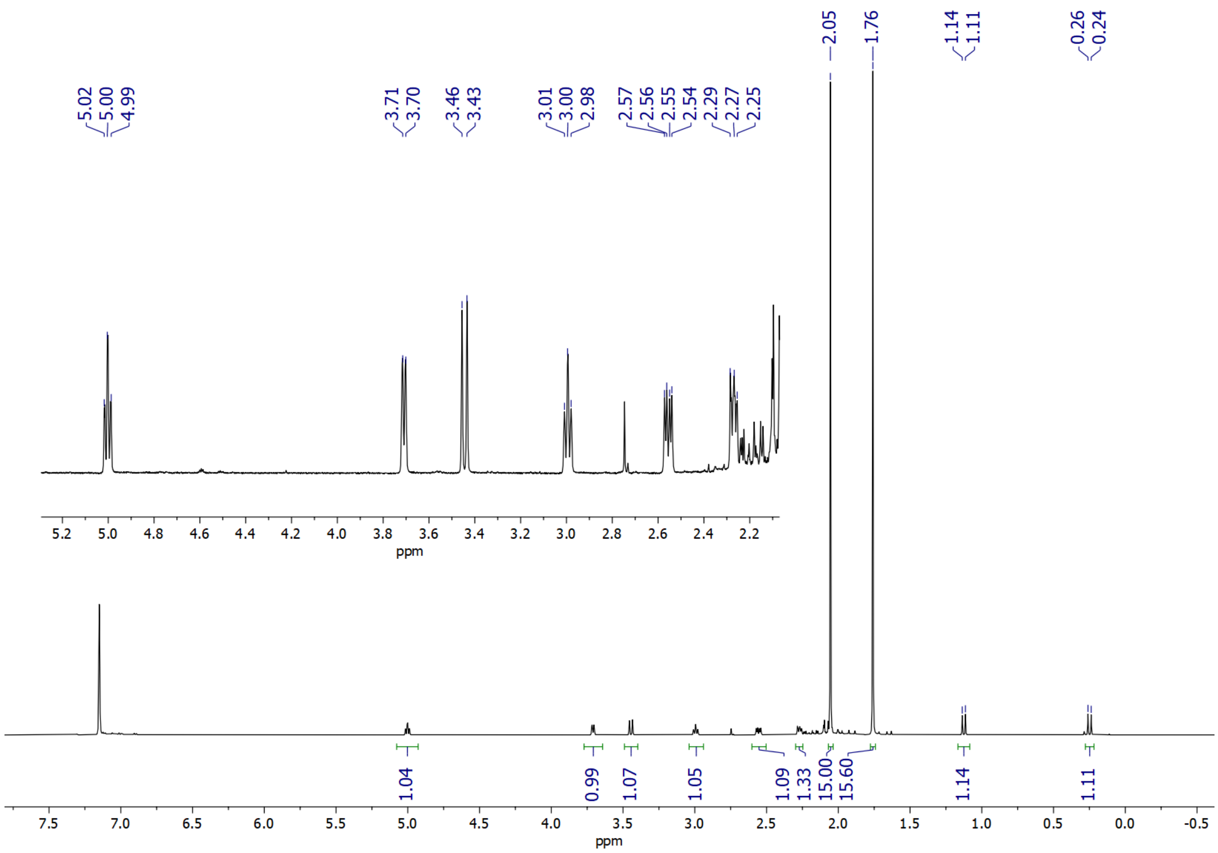

Figure S24. ^{1}H NMR spectrum of compound [Ta(η^5-C_5Me_5)(μ-CH_2-C_6H_6)(μ-S)$_2$] (**7**) in C_6D_6 (500 MHz).

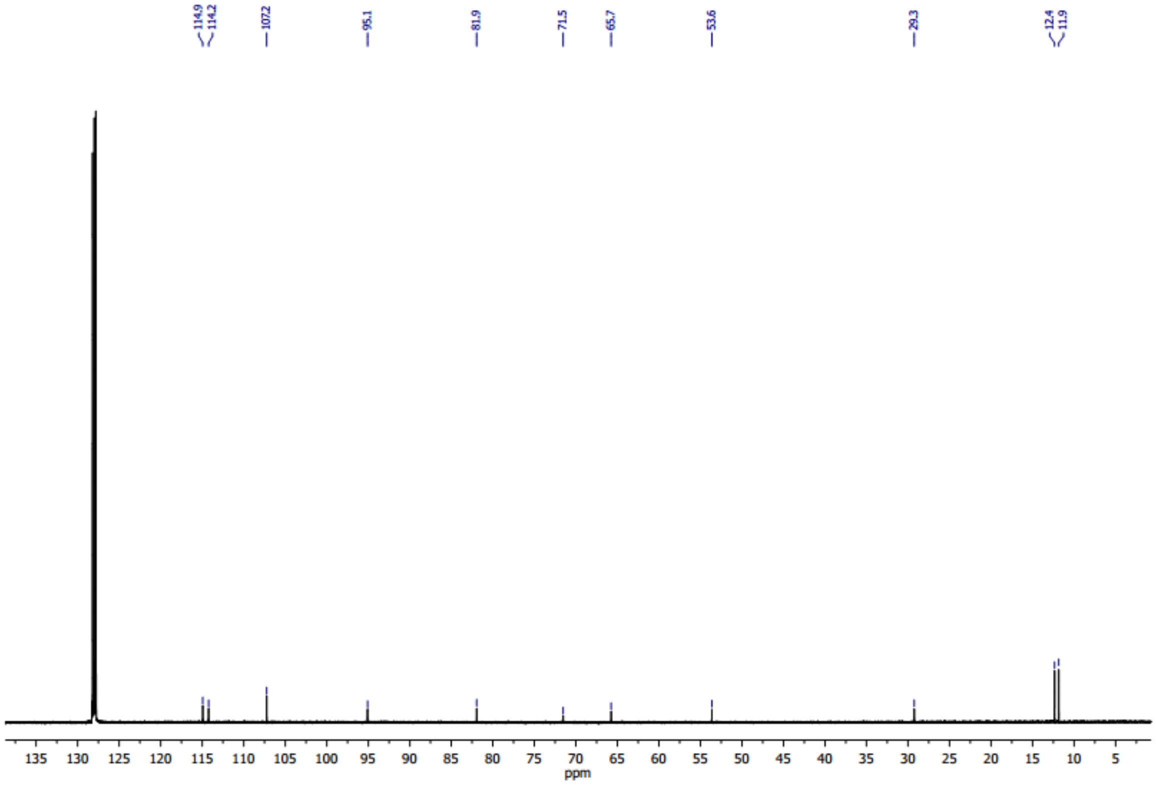

Figure S25. ^{13}C NMR spectrum of compound [Ta(η^5-C_5Me_5)(μ-CH_2-C_6H_6)(μ-S)$_2$] (**7**) in C_6D_6 (125 MHz).

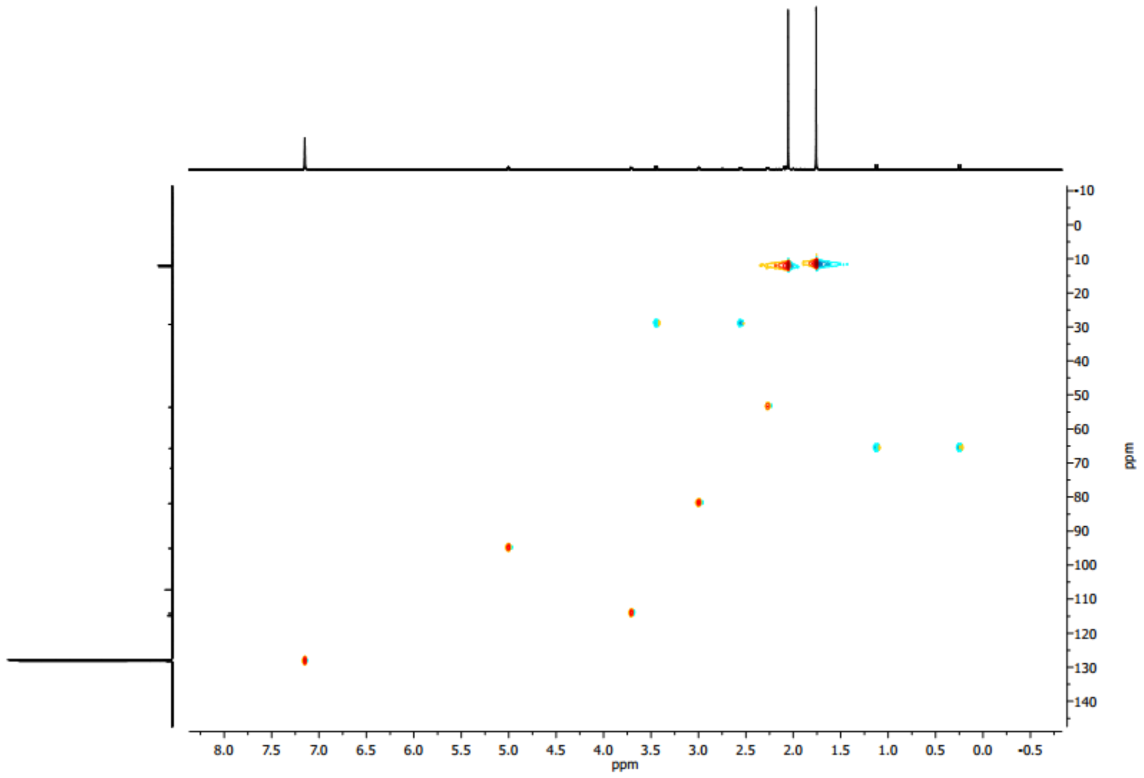

Figure S26. g-HSQC NMR spectrum of compound [Ta(η^5-C_5Me_5)(μ-CH_2-C_6H_6)(μ-S)$_2$] (7) in C_6D_6 (500 MHz).

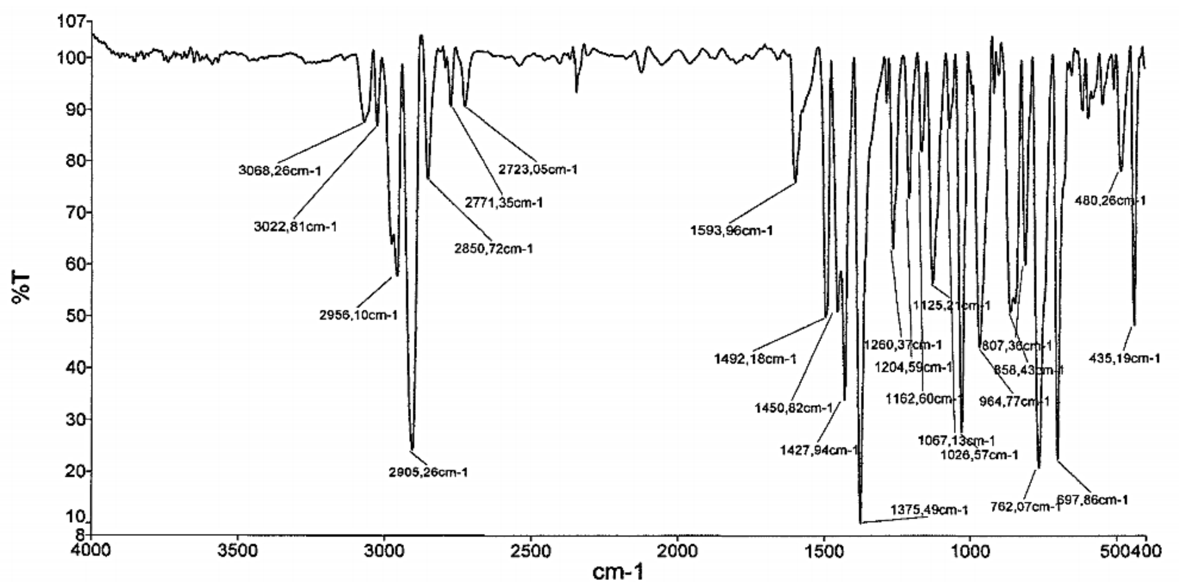

Figure S27. IR spectrum (KBr) for complex [Ta(η^5-C_5Me_5)(μ-CH_2-C_6H_6)(μ-S)$_2$] (7).

Computational Study

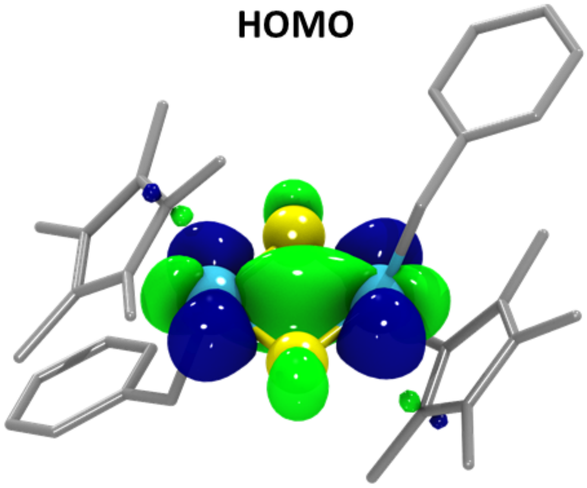

Figure S28. HOMO molecular orbital of complex **2**, consisting in a σ-bonding combination between d orbitals centered at each tantalum atom.

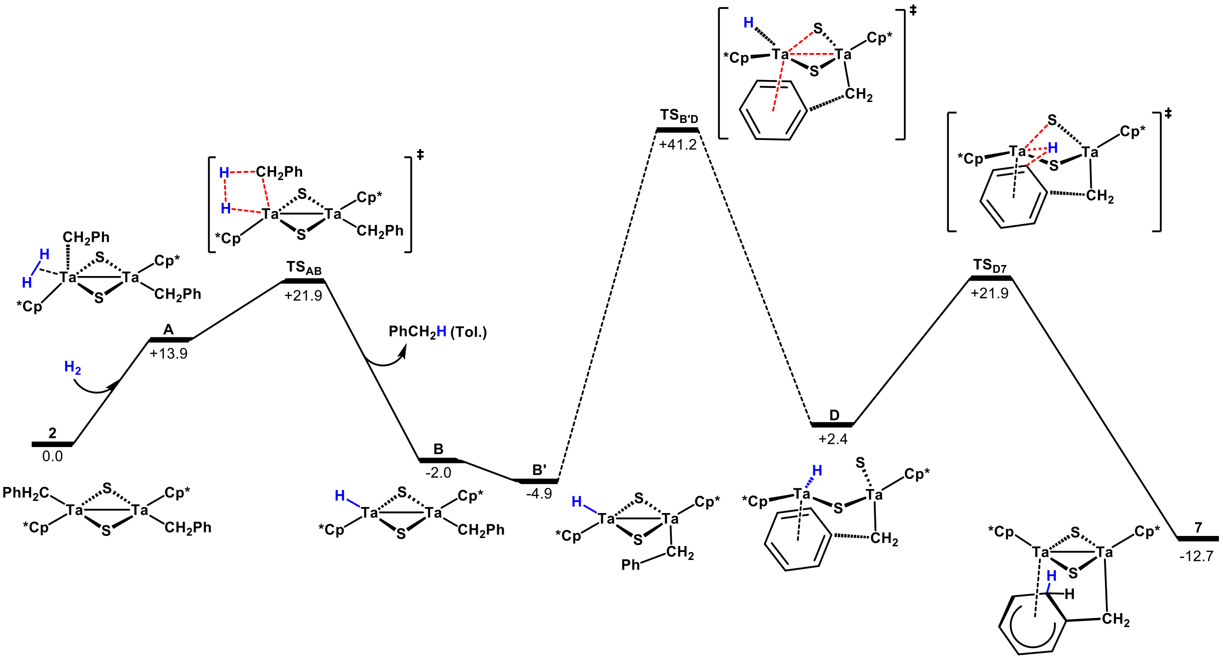

Figure S29. Gibbs free energy profile (kcal·mol^{-1}) of an alternative pathway for the formation of complex **7** through the cleavage of a S-Ta bond, obtaining a Ta(III)-Ta(V) intermediate (**D**) which appears to be energetically unfeasible ($\Delta G^{\ddagger}_{overall} = 46.1$ kcal·mol^{-1}).

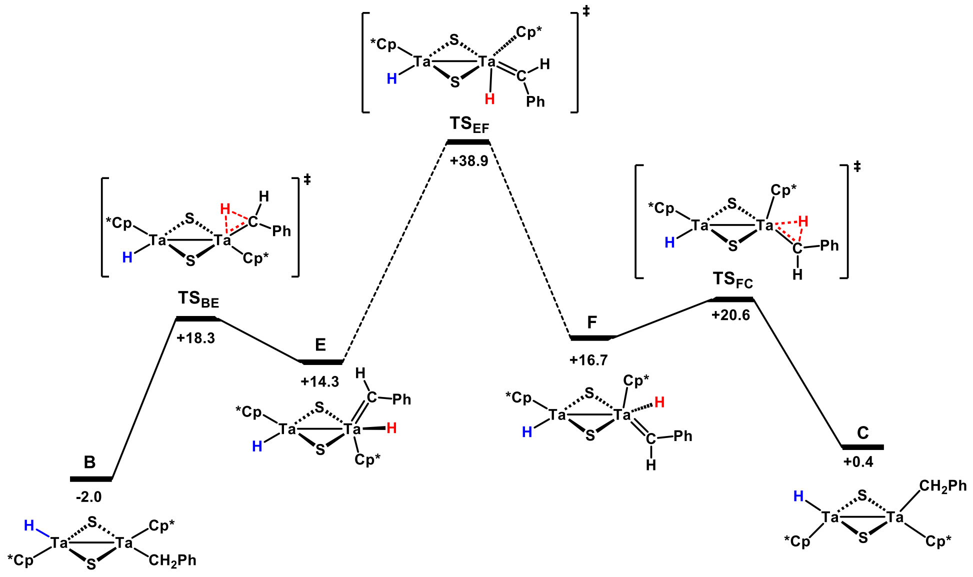

Figure S30. Gibbs free energy profile (kcal·mol^{-1}) of an alternative pathway for the transformation of **B** to **C** through the formation of an alkylidene intermediate which is able to rotate tantalum ligands. This mechanism was discarded due to the high overall energetic barrier ($\Delta G^{\ddagger}_{overall}$ = 40.9 kcal·mol^{-1}).
